# Supplementary material for: Design and Synthesis of Novel Podophyllotoxins Hybrids and the Effects of Different Functional Groups on Cytotoxicity
Source: Molecules. 2021 Dec 30;27(1):220. doi: 10.3390/molecules27010220 (PMC8746343; doi:10.3390/molecules27010220)

Supplementary Materials

Design and Synthesis of Novel Podophyllotoxins Hybrids and the Effects of Different Functional Groups on Cytotoxicity

Zhongtao Yang [1,2,3], Zitong Zhou [1], Xiai Luo [1,2,3], Xiaoling Luo [3], Hui Luo [1,2,*], Lianxiang Luo [1,*] and Weiguang Yang [1,2,*]

[1] The Marine Biomedical Research Institute, Guangdong Medical University, Zhanjiang 524023, China; yangzt@gdmu.edu.cn (Z.Y.); zzt15766229745@163.com (Z.Z.); luoxiai83@163.com (X.L.)

[2] The Marine Biomedical Research Institute of Guangdong Zhanjiang, Zhanjiang 524023, China

[3] School of Basic Medical Sciences, Guangxi Medical University, Nanning 530021, China; luoxiaoling@gxmu.edu.cn

* Correspondence: luohui@gdmu.edu.cn (H.L.); luolianxiang321@163.com (L.L.); yangwg@gdmu.edu.cn (W.Y.).

Table of Contents

1. General Information……………………………………………………………..…S2

2. The structures of starting materials **1a-1b**, **2a-2m** and **3a-3j**……………….….…S3

3. Copies of NMR Spectra…………………………………………………….....…S4

1. General Information

^{1}H NMR spectrum were recorded on a Bruker DPX 400 MHz spectrometer in $CDCl_3$. Chemical shifts were reported in ppm with the internal TMS signal at 0.0 ppm as a standard. The spectra are interpreted as: s, singlet; bs, broad singlet; d, doublet; t, triplet; q, quartet; m, multiplet; dd, double doublet; ddd, double double doublet; dt, double triplet; ddt, double double triplet; tt, triple triplet, td, triple doublet; coupling constant(s) *J* are reported in Hz and relative integrations are reported. ^{13}C NMR (100 MHz) spectrum were recorded on a Bruker DPX 400 MHz spectrometer in $CDCl_3$. Chemical shifts were reported in ppm with the internal chloroform signal at 77.16 ppm as a standard; additionally, using others residual nondeuterated solvent as internal standard (CD_3OD δ 3.31 for ^{1}H and 49.00 ppm for ^{13}C; $(CD_3)_2SO$: δ 2.50 for ^{1}H and 39.52 ppm for ^{13}C; $(CD_3)_2CO$: δ 2.05 for ^{1}H and 29.84 ppm for ^{13}C). Melting points were obtained in open capillary tubes using SGW X-4 micro melting point apparatus which were uncorrected. Mass spectrum were recorded on TOF mass spectrometer. All reagents and starting materials were purchased from Adamas-beta and other suppliers and used without further purification. Methyl cyanide, dichloromethane (DCE) and dichloromethane (DCM) was dried by distillation over CaH_2.

2. The structures of starting materials 1a-1b, 2a-2m and 3a-3j

Scheme S1 Structures of the starting materials **1a-1b**.

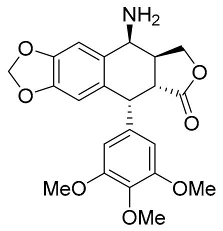

1a

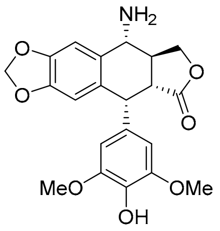

1b

Scheme S2 Structures of the starting materials **2a-2m**.

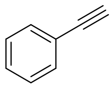

2a

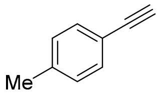

2b

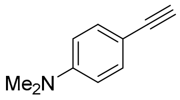

2c

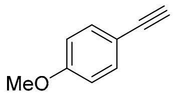

2d

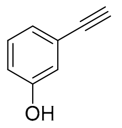

2e

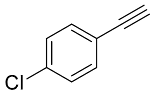

2f

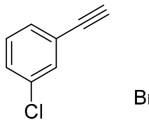

2g

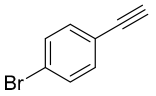

2h

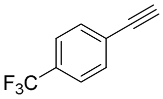

2i

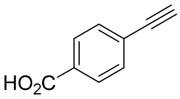

2j

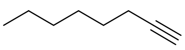

2k

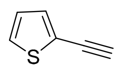

2l

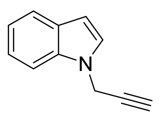

2m

Scheme S3 Structures of the starting materials **3a-3j**.

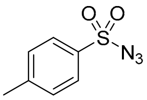

3a

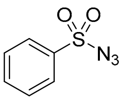

3b

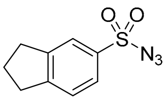

3c

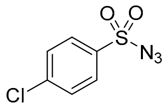

3d

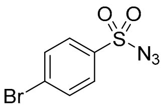

3e

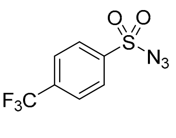

3f

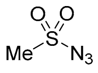

3g

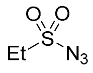

3h

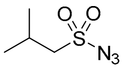

3i

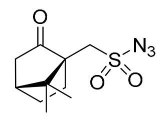

3j

3. Copies of NMR spectra

Figure S1 ^{1}H NMR spectrum of compound **4a** ($CDCl_3$)

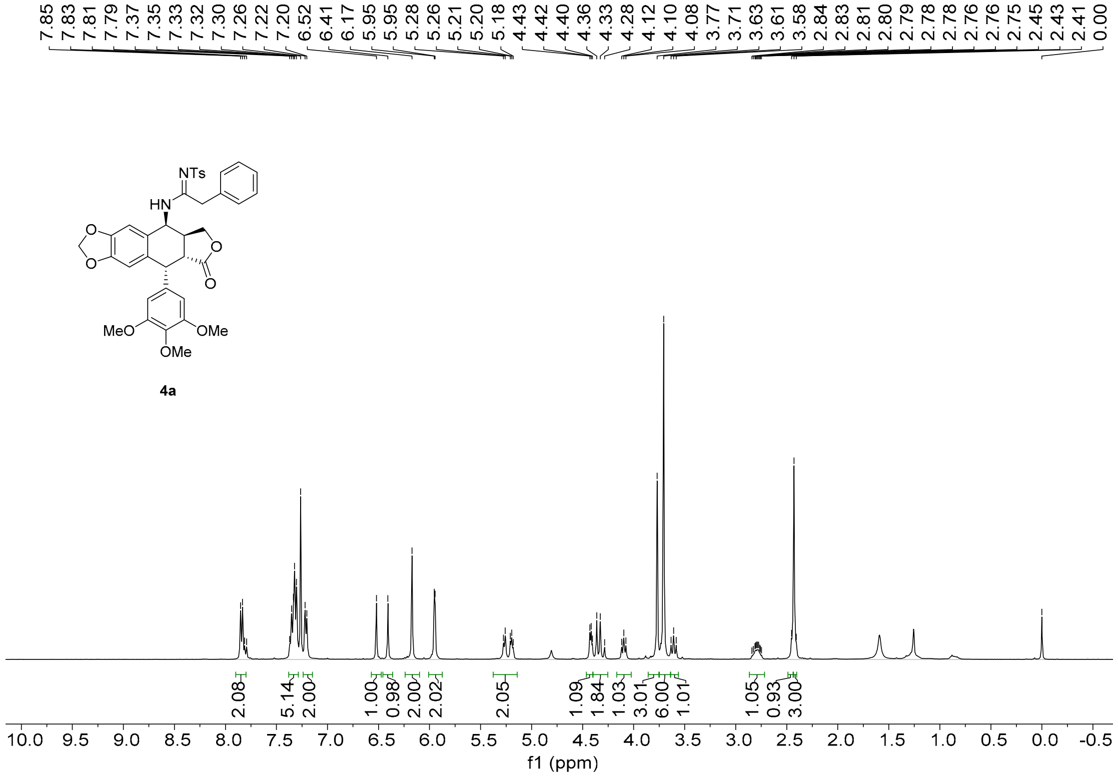

Figure S2 ^{13}C NMR spectrum of compound **4a** ($CDCl_3$)

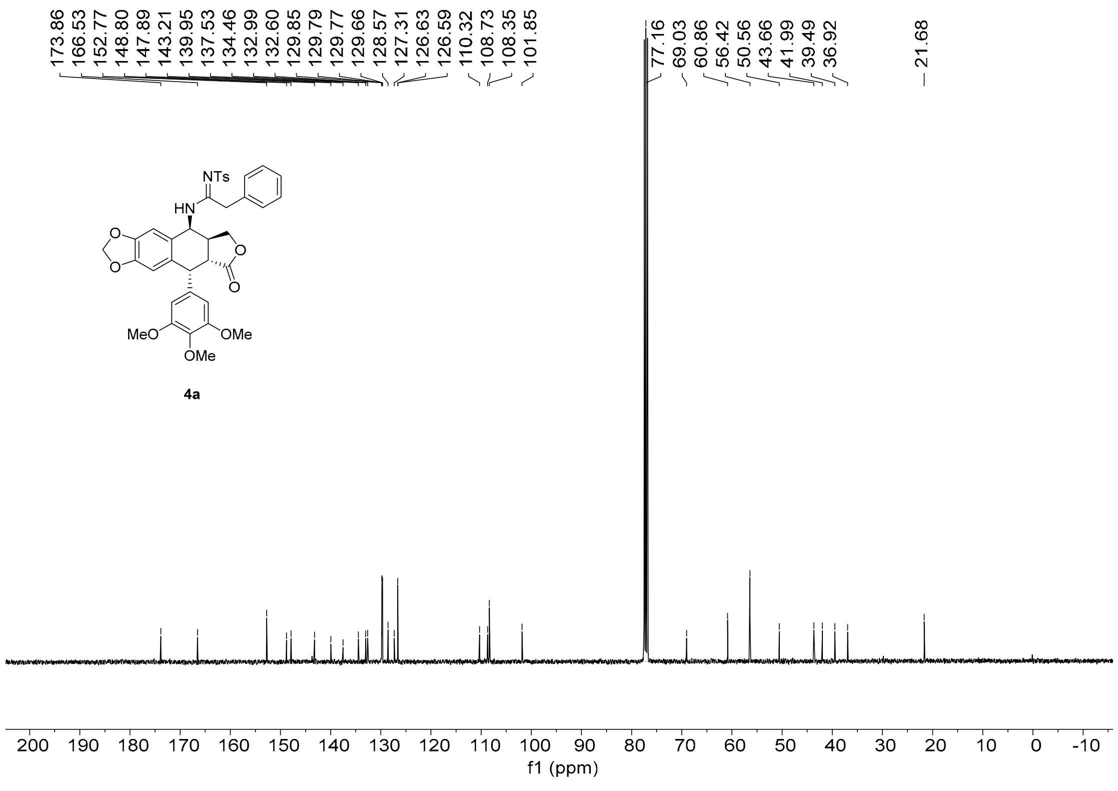

Figure S3 ^{1}H NMR spectrum of compound **4b** ($CDCl_3$)

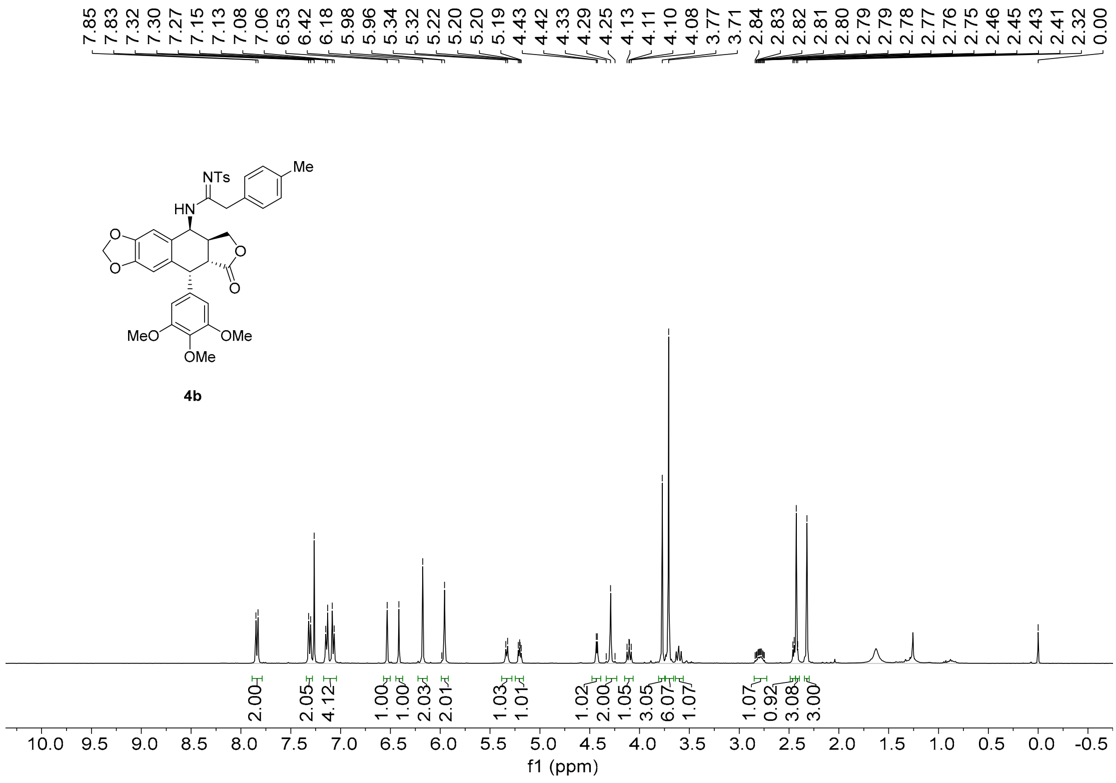

Figure S4 ^{13}C NMR spectrum of compound **4b** ($CDCl_3$)

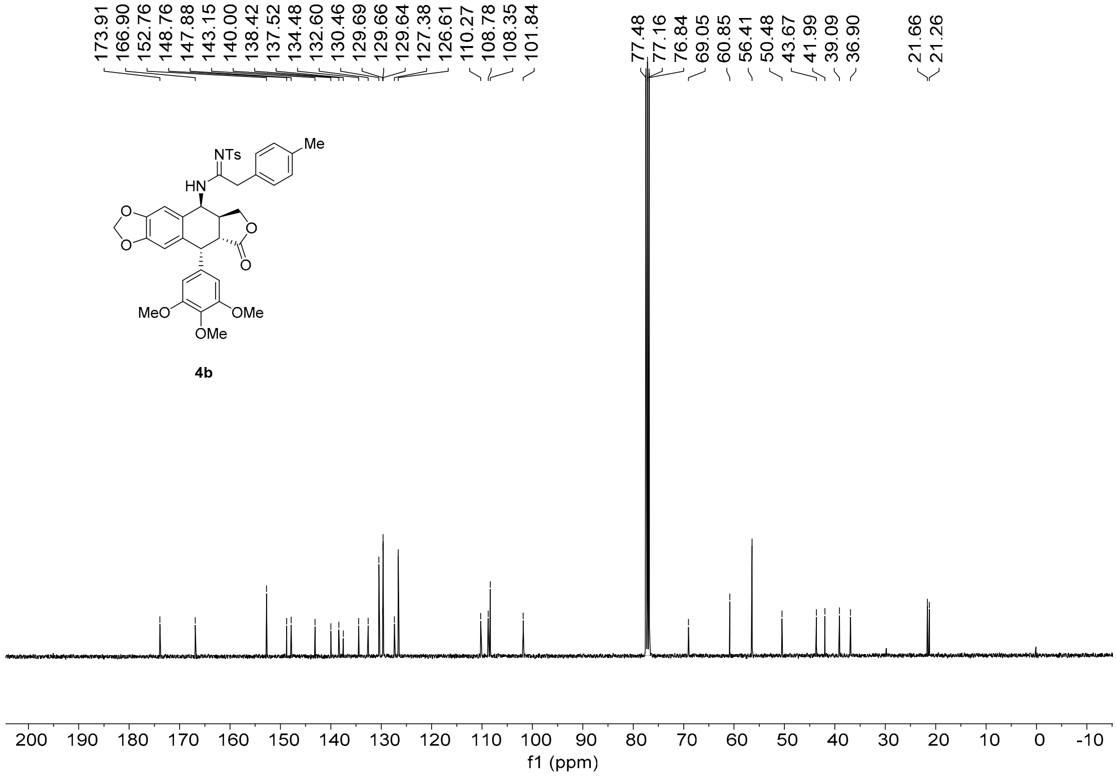

Figure S5 ^{1}H NMR spectrum of compound **4c** ($CDCl_3$)

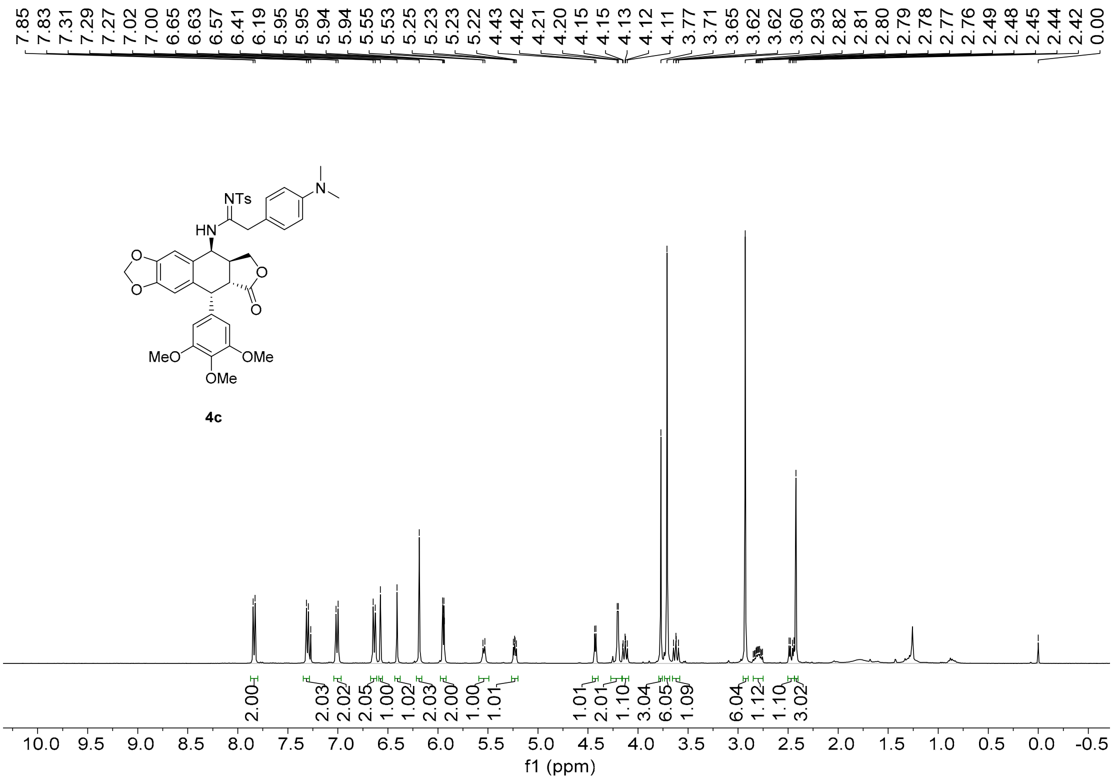

Figure S6 ^{13}C NMR spectrum of compound **4c** ($CDCl_3$)

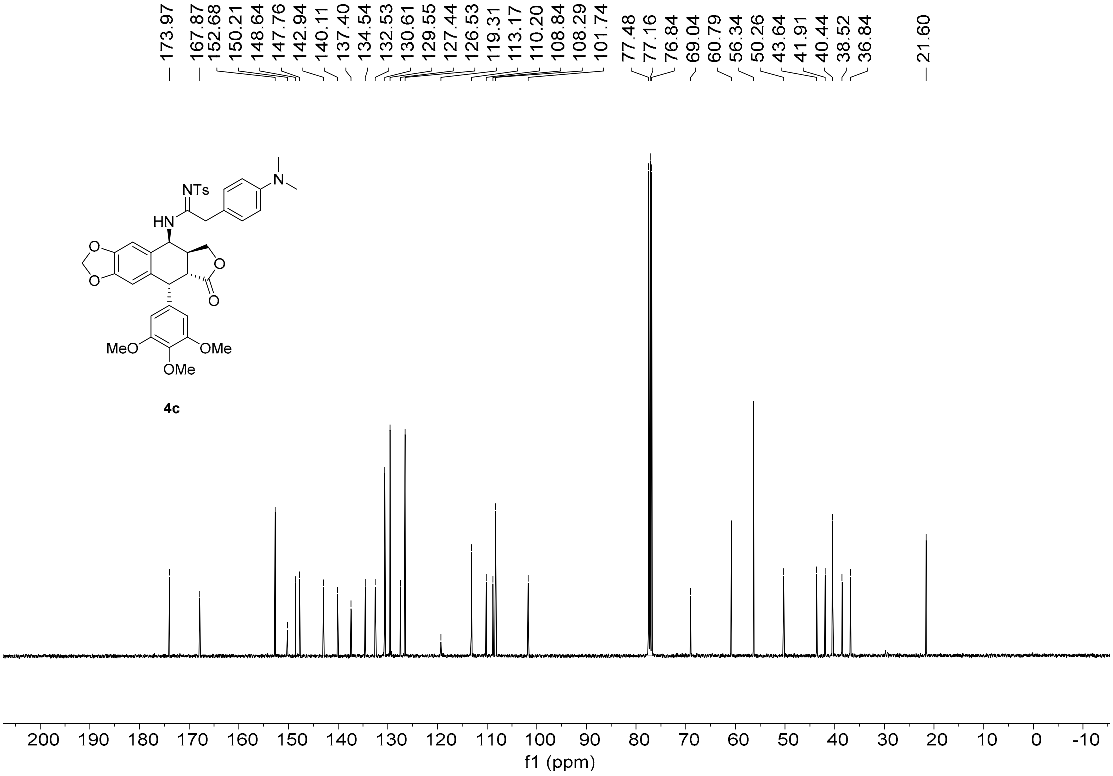

Figure S7 ^{1}H NMR spectrum of compound **4d** ($CDCl_3$)

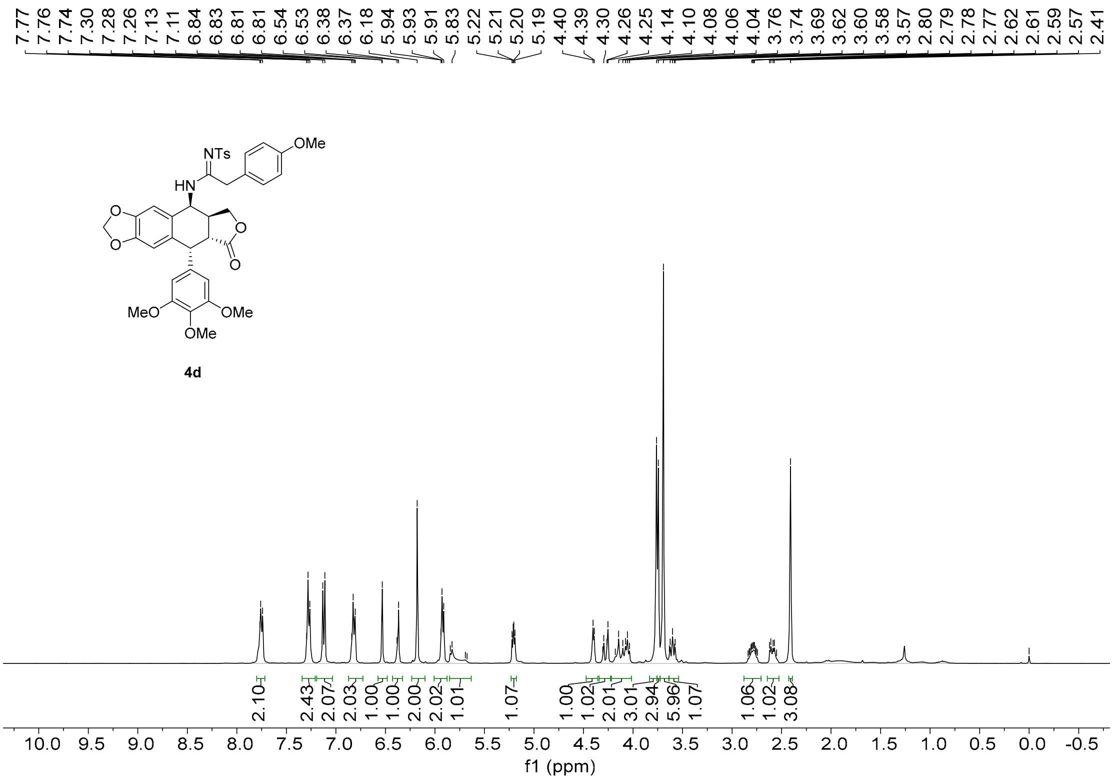

Figure S8 ^{13}C NMR spectrum of compound **4d** ($CDCl_3$)

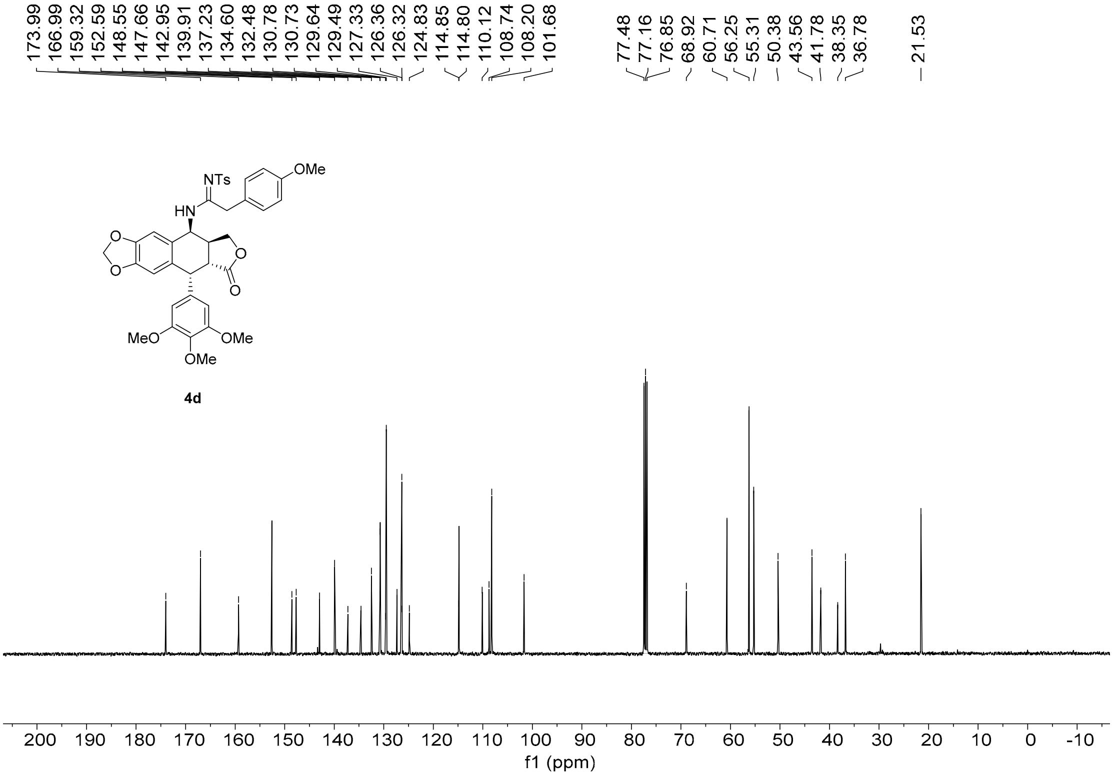

Figure S9 ^{1}H NMR spectrum of compound **4e** ($CDCl_3$)

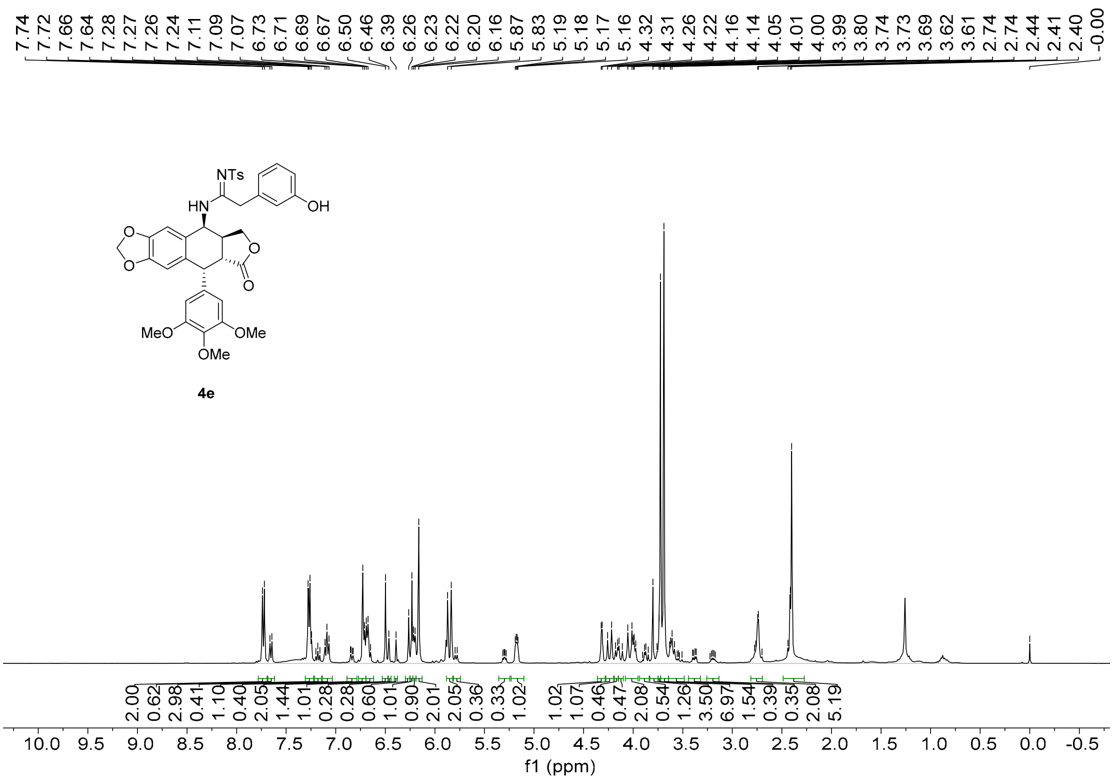

Figure S10 ^{13}C NMR spectrum of compound **4e** ($CDCl_3$)

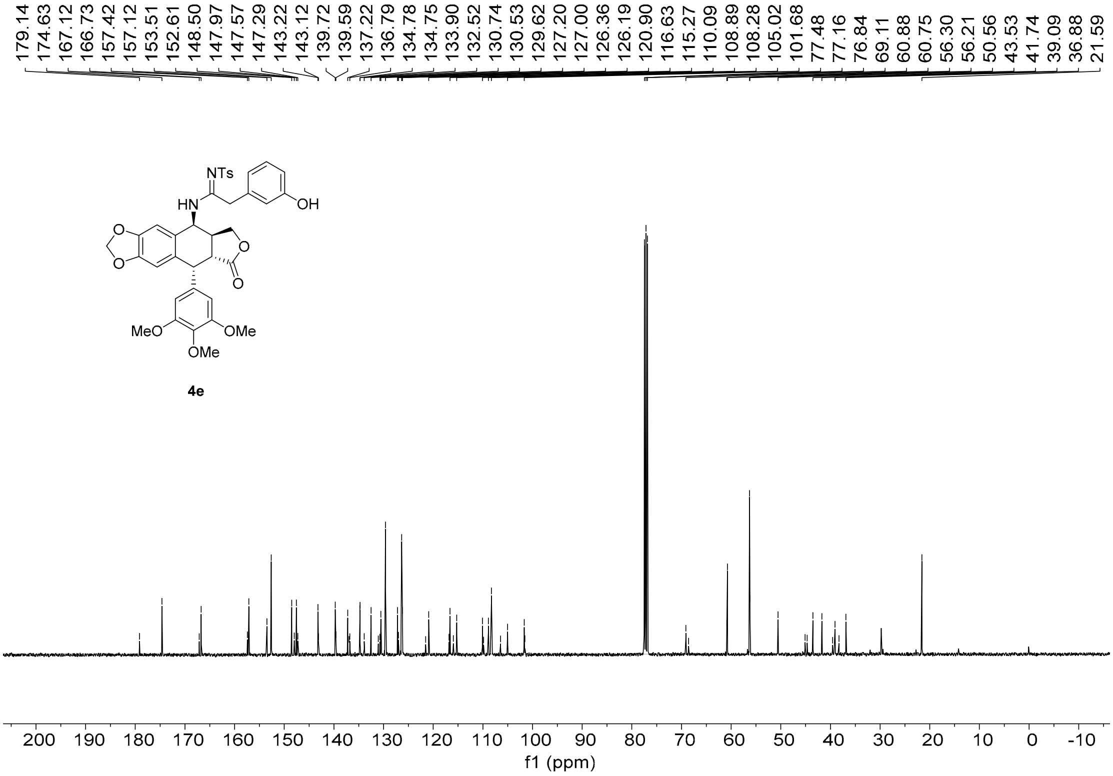

Figure S11 ^{1}H NMR spectrum of compound **4f** ($CDCl_3$)

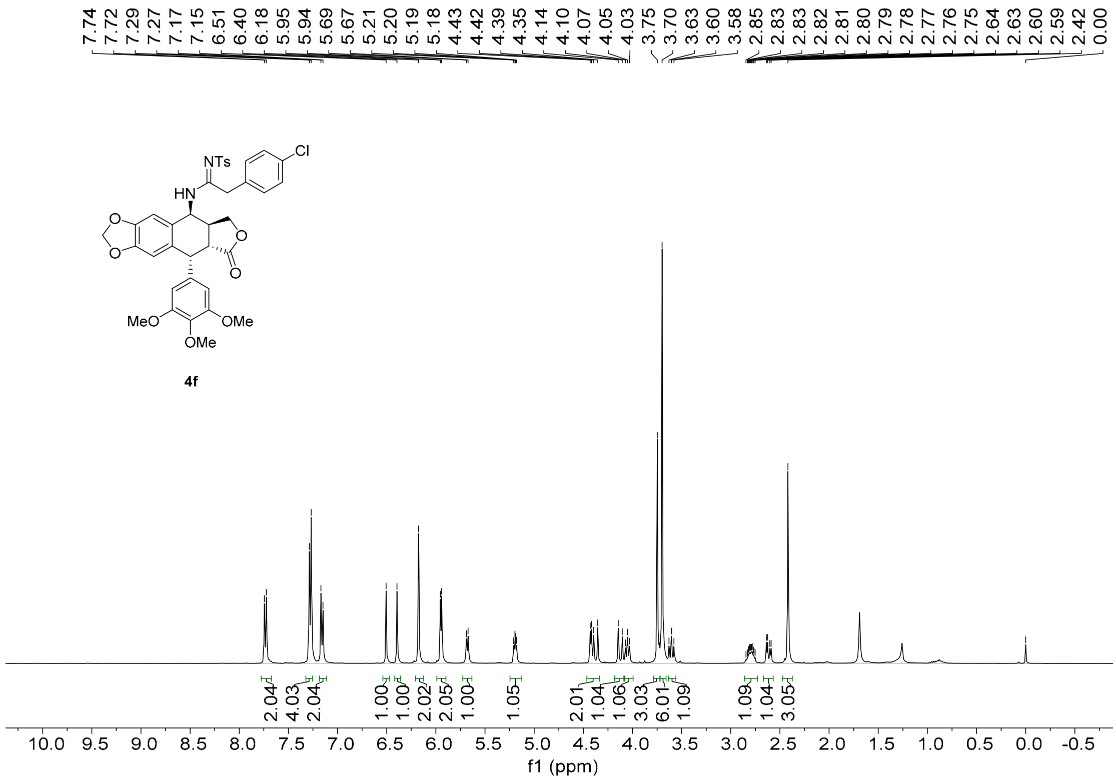

Figure S12 ^{13}C NMR spectrum of compound **4f** ($CDCl_3$)

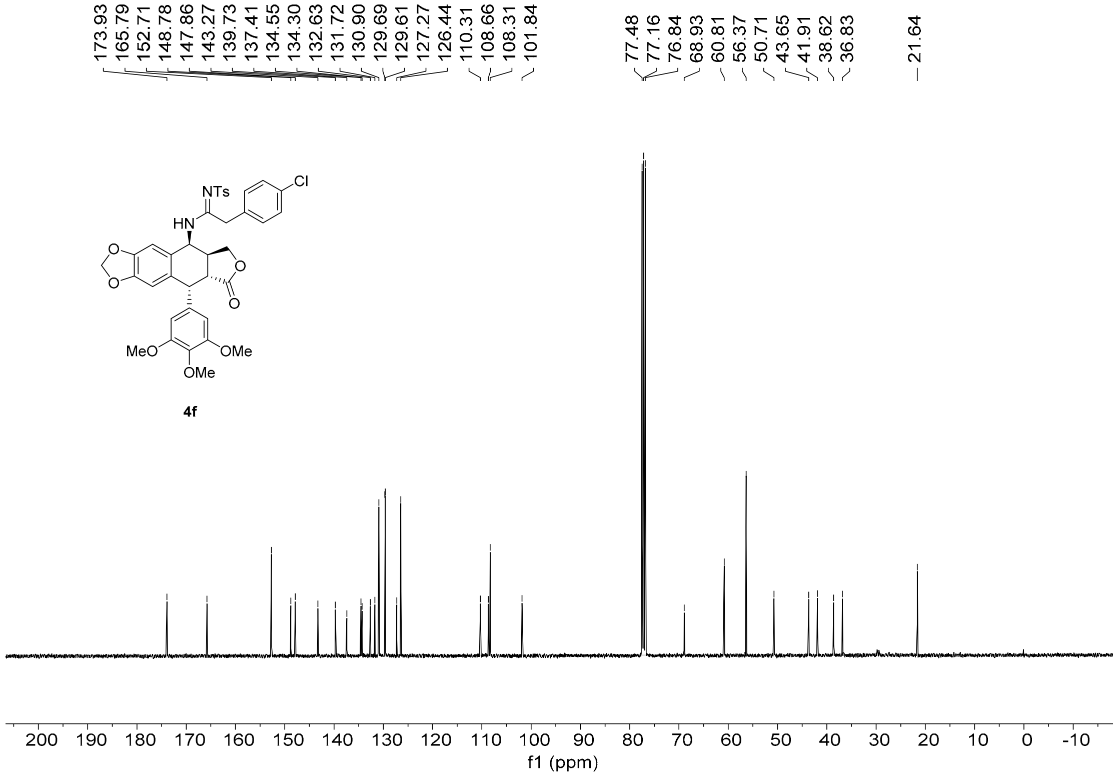

Figure S13 ^{1}H NMR spectrum of compound **4g** ($CDCl_3$)

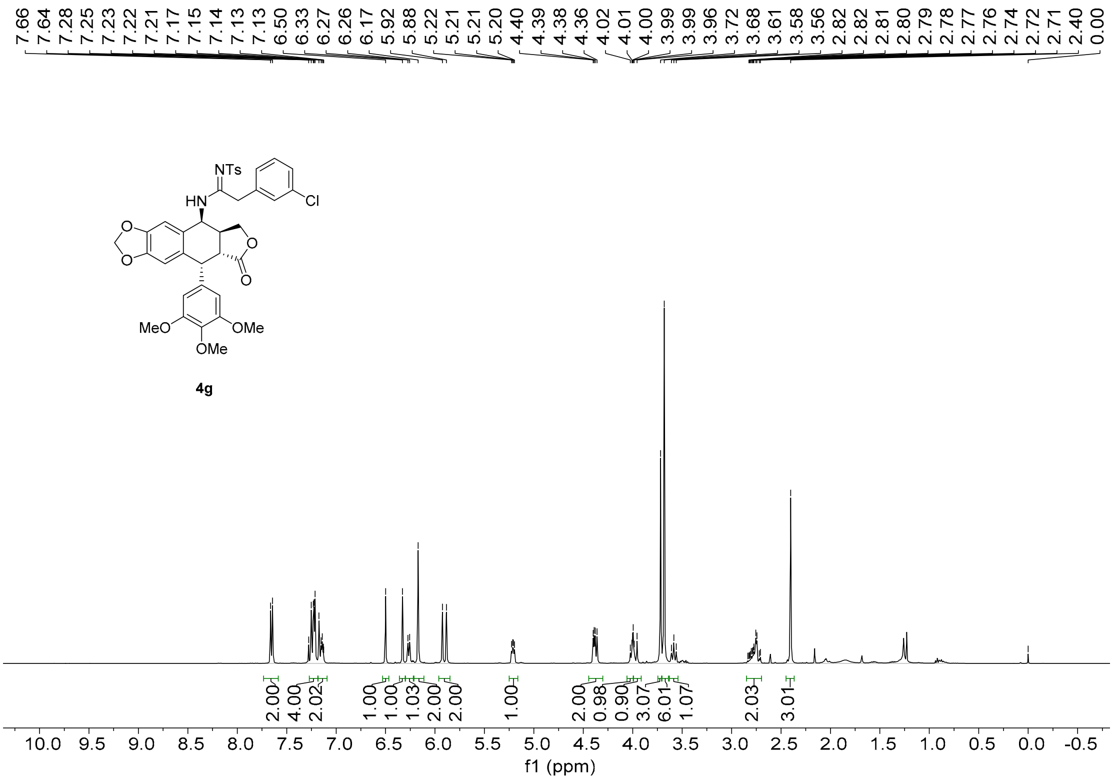

Figure S14 ^{13}C NMR spectrum of compound **4g** ($CDCl_3$)

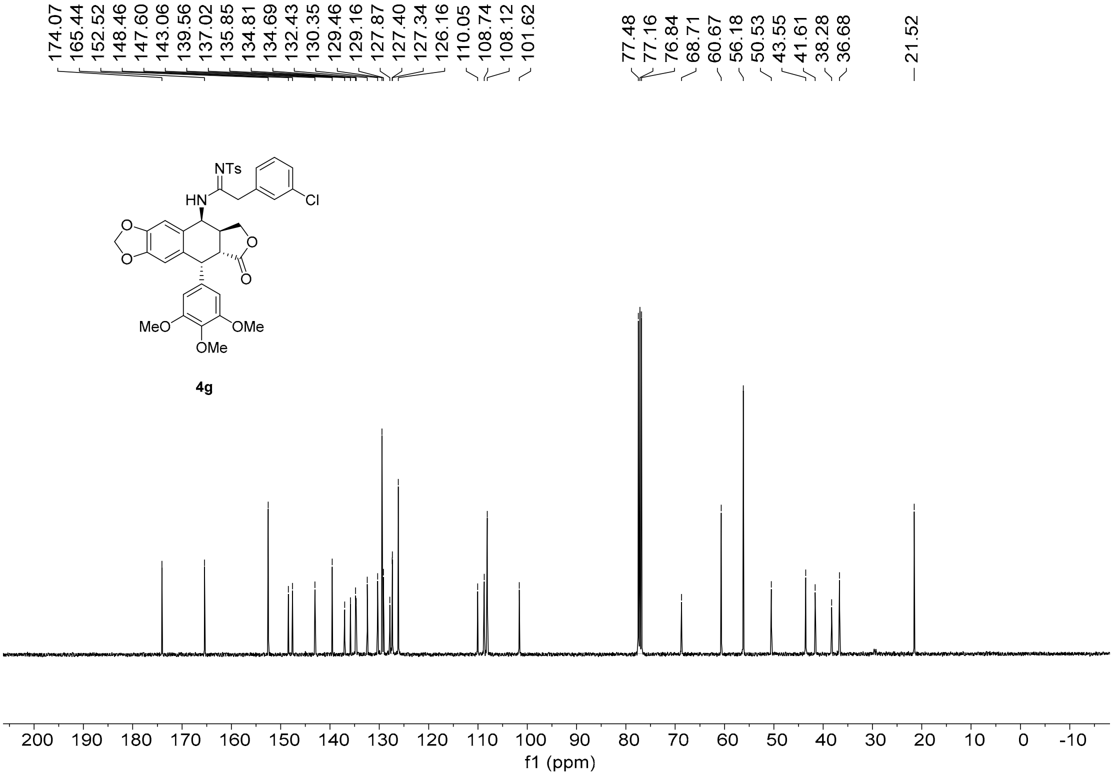

Figure S15 ^{1}H NMR spectrum of compound **4h** ($CDCl_3$)

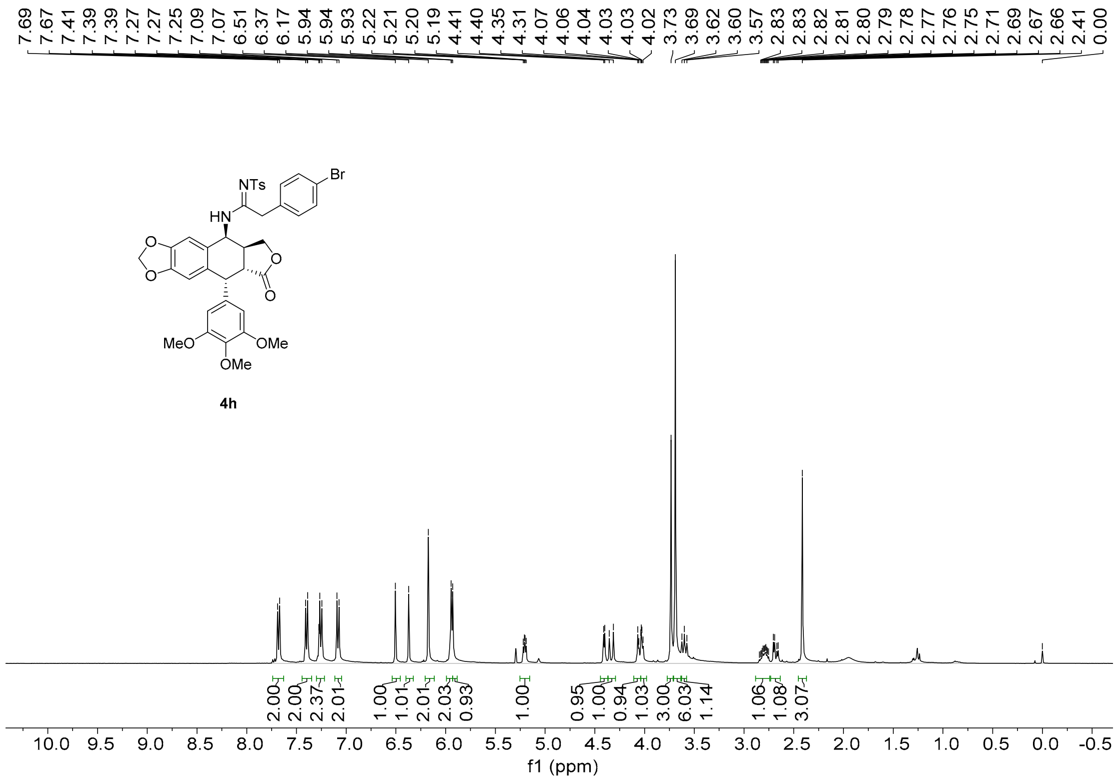

Figure S16 ^{13}C NMR spectrum of compound **4h** ($CDCl_3$)

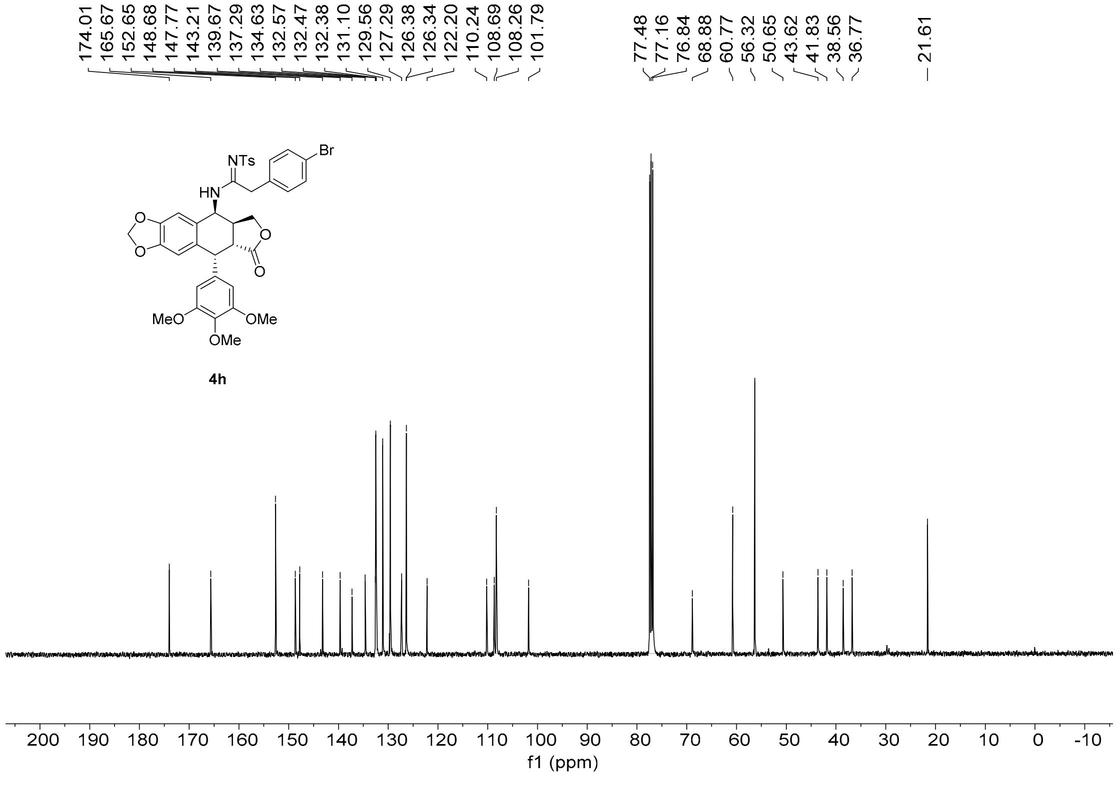

Figure S17 ^{1}H NMR spectrum of compound **4i** ($CDCl_3$)

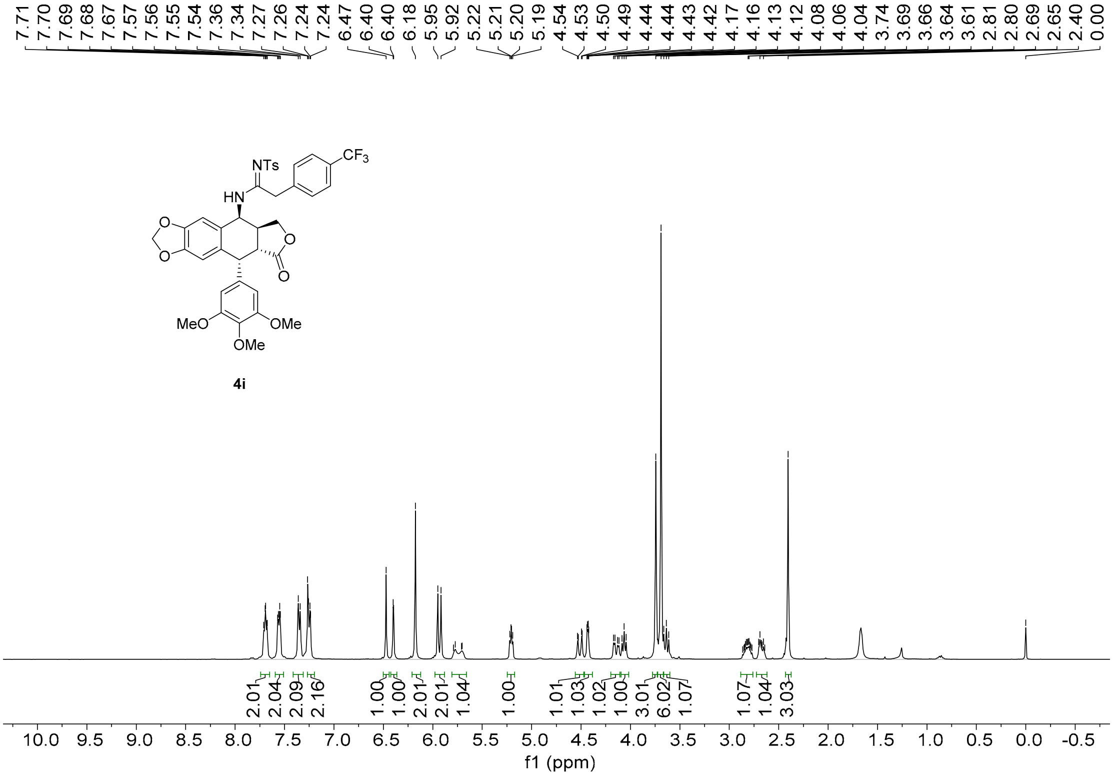

Figure S18 ^{13}C NMR spectrum of compound **4i** ($CDCl_3$)

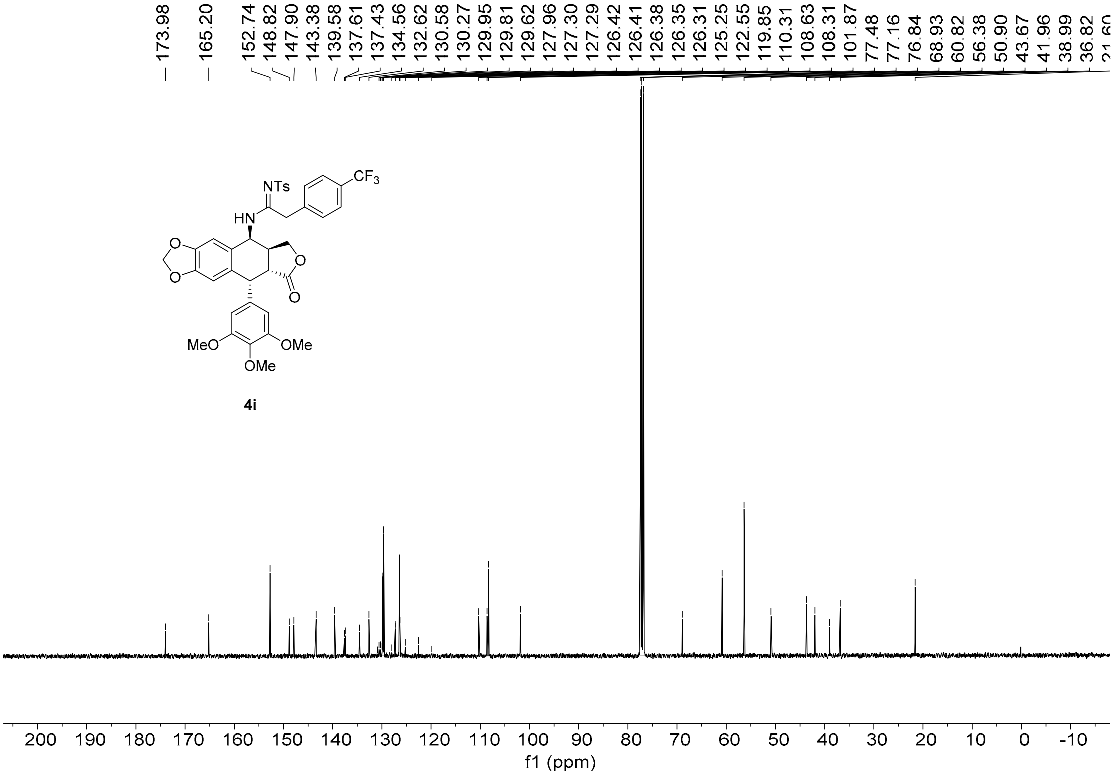

Figure S19 ^{1}H NMR spectrum of compound **4j** ($(CD_3)_2CO$)

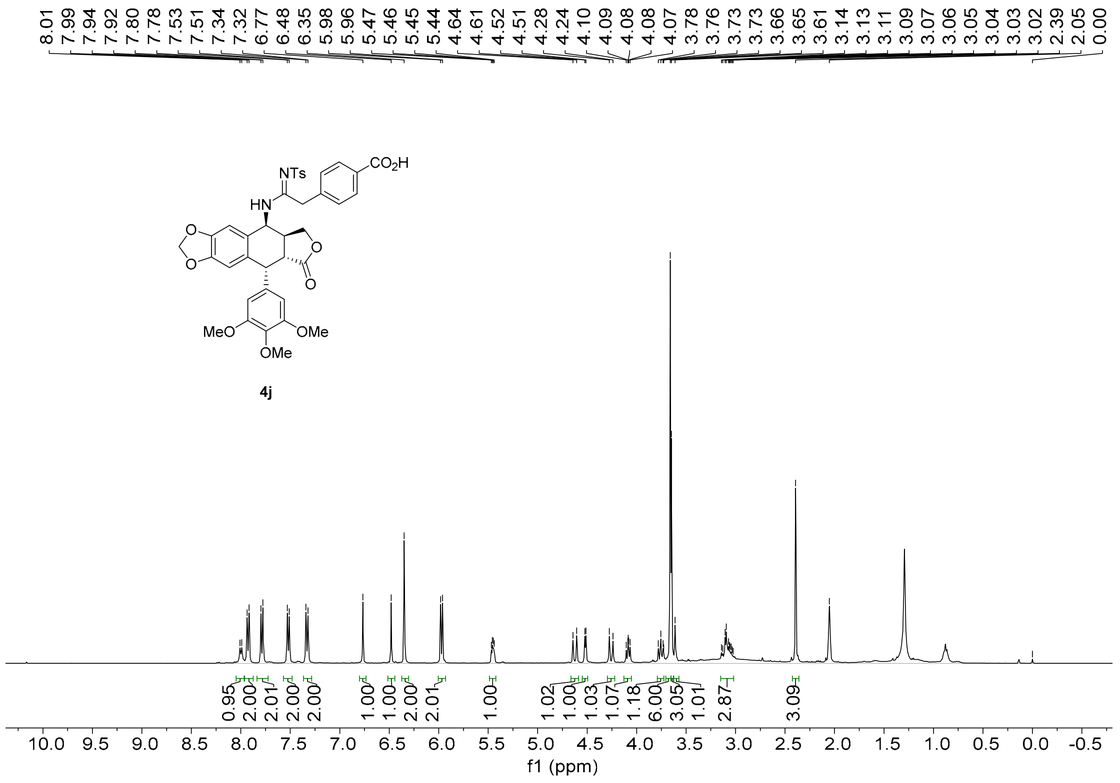

Figure S20 ^{13}C NMR spectrum of compound **4j** ($(CD_3)_2CO$)

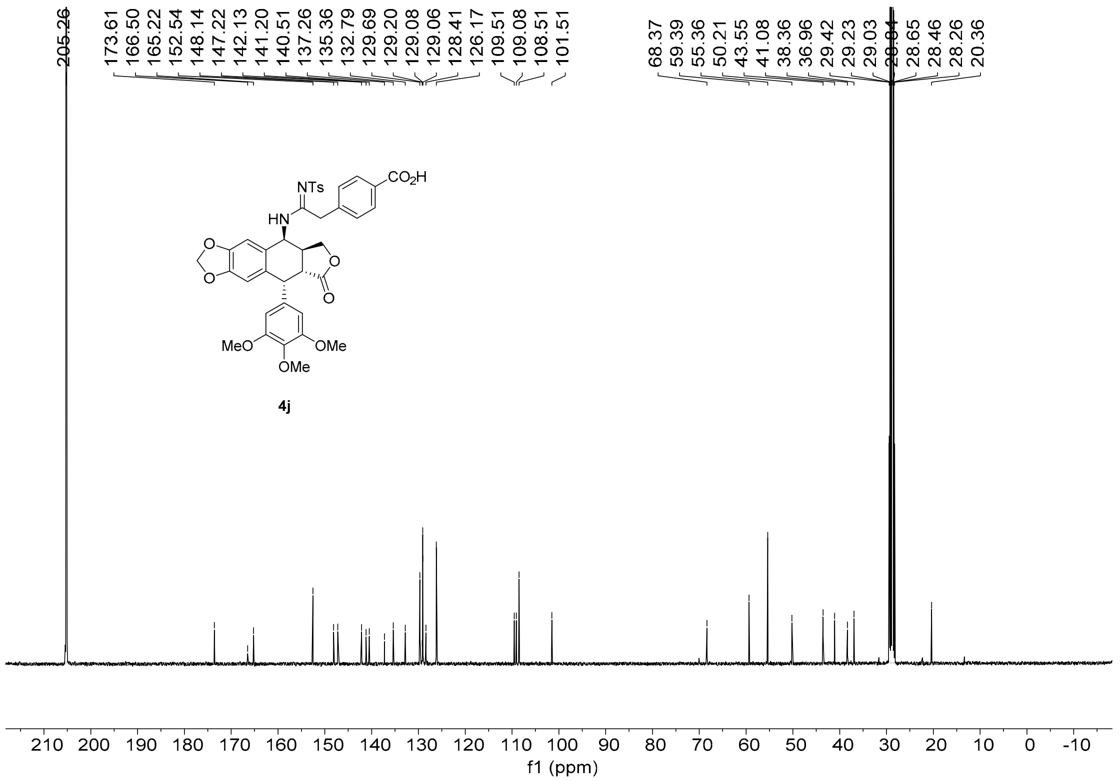

Figure S21 ^{1}H NMR spectrum of compound **4k** ($CDCl_3$)

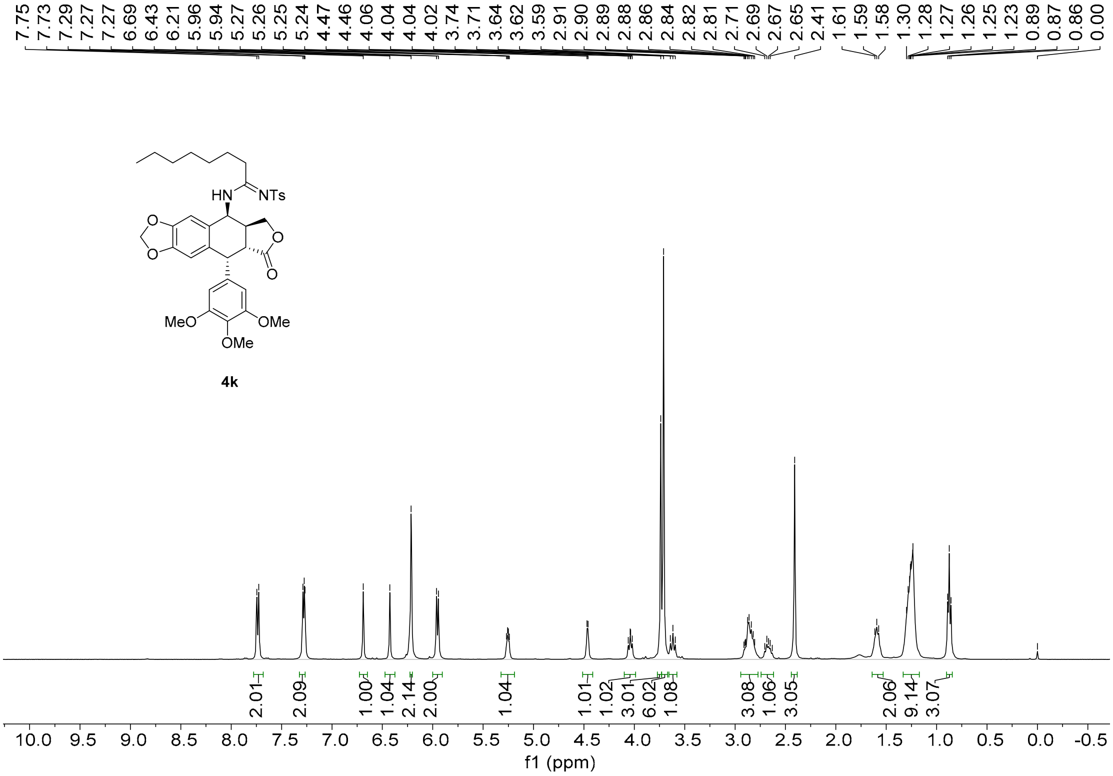

Figure S22 ^{13}C NMR spectrum of compound **4k** ($CDCl_3$)

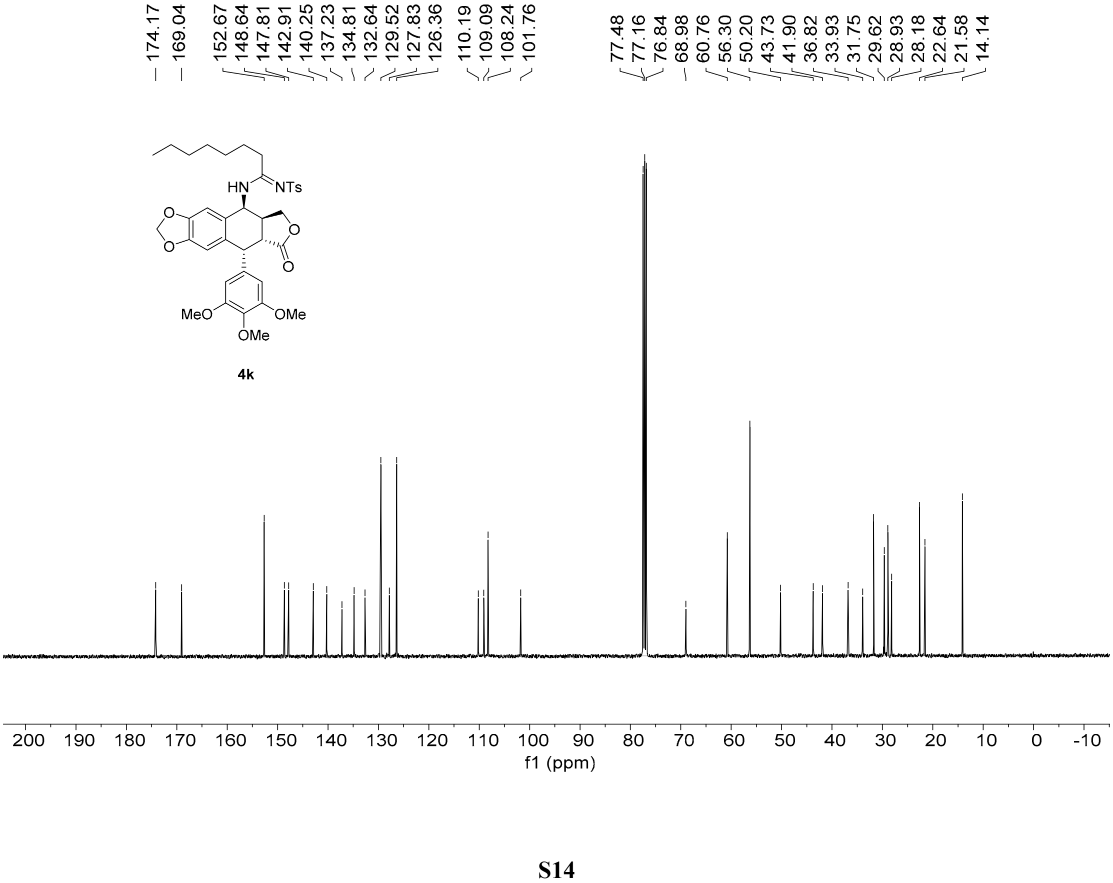

Figure S23 ^{1}H NMR spectrum of compound **4l** ($CDCl_3$)

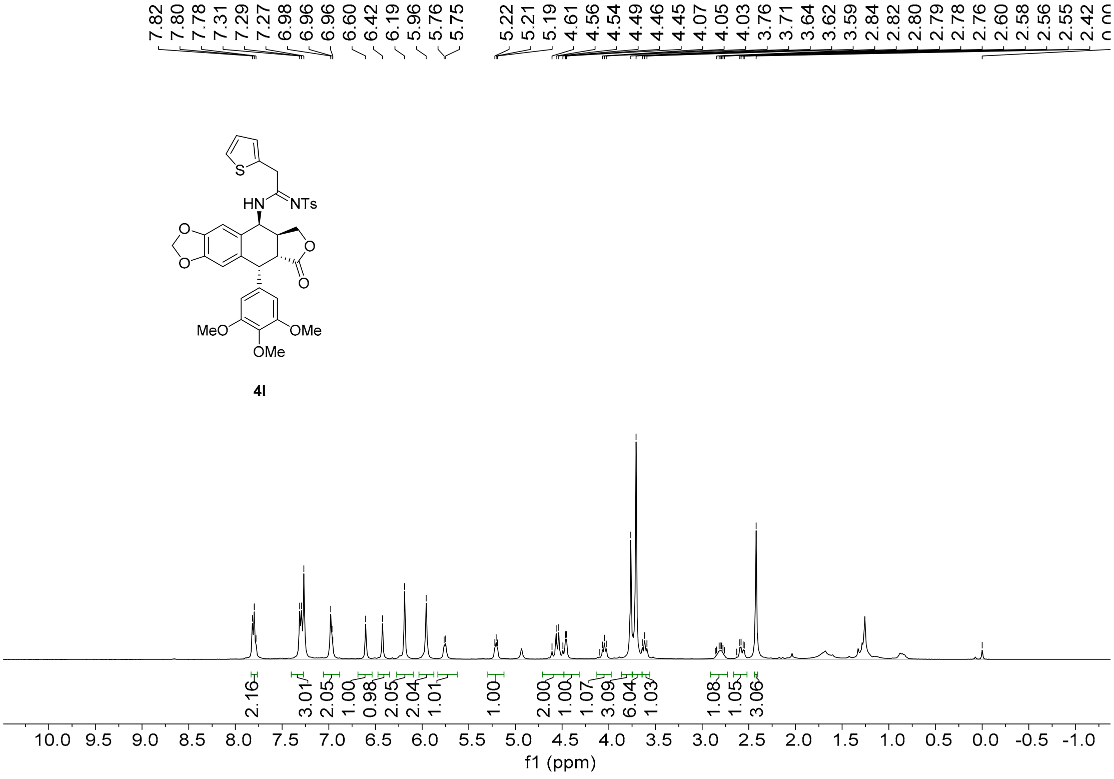

Figure S24 ^{13}C NMR spectrum of compound **4l** ($CDCl_3$)

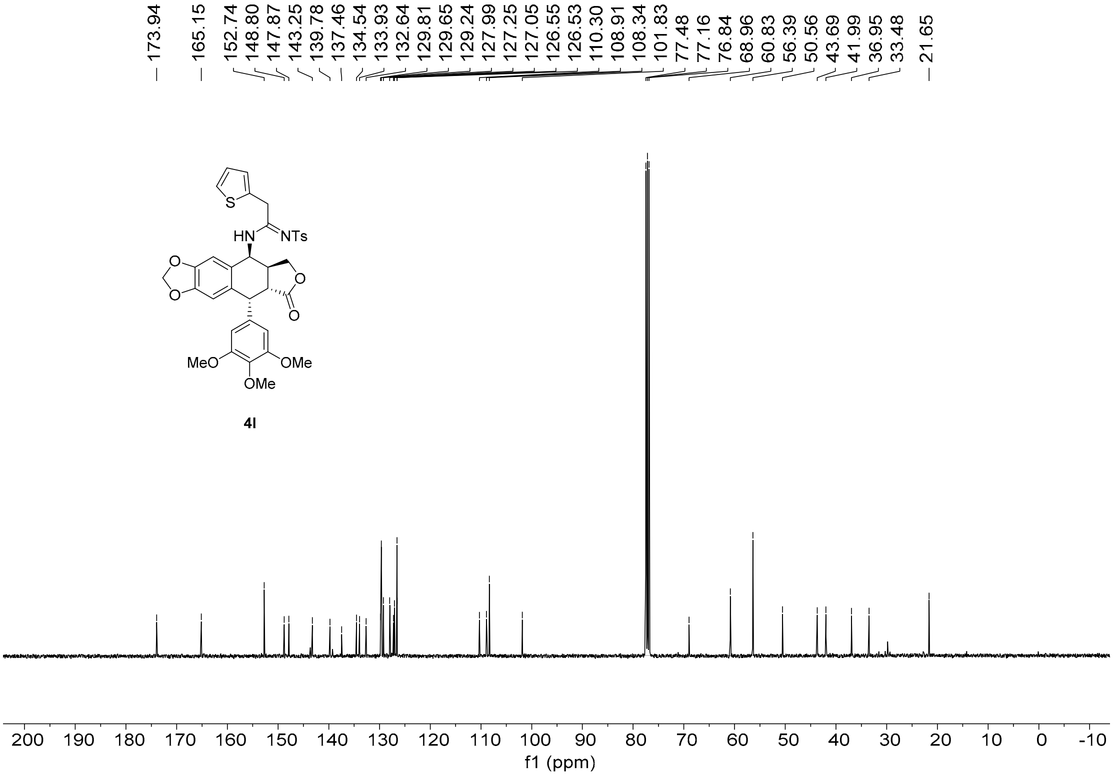

Figure S25 ^{1}H NMR spectrum of compound **4m** ($CDCl_3$)

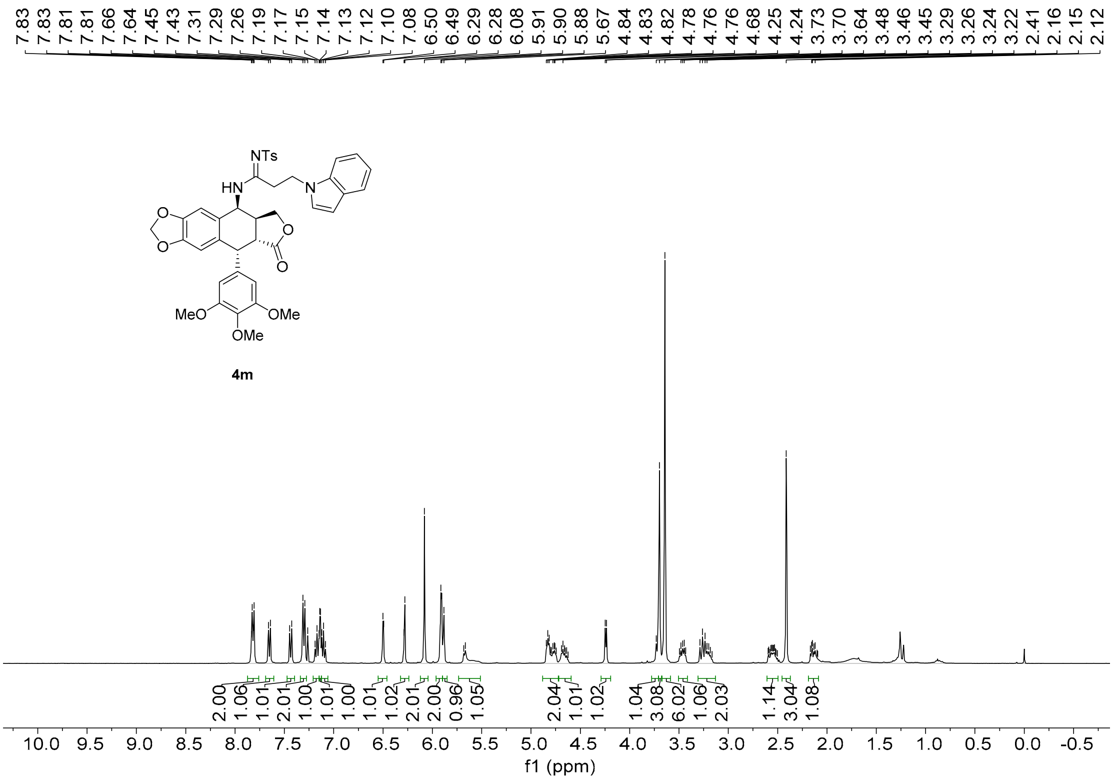

Figure S26 ^{13}C NMR spectrum of compound **4m** ($CDCl_3$)

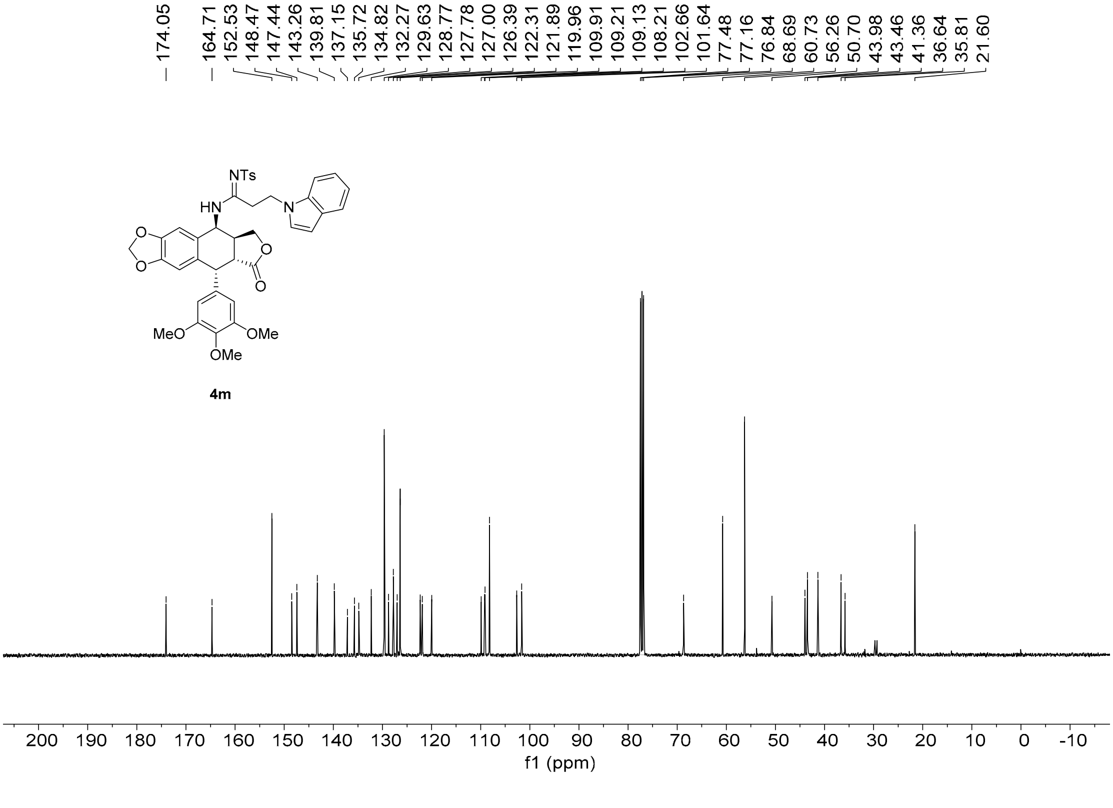

Figure S27 ^{1}H NMR spectrum of compound **4n** ($CDCl_3$)

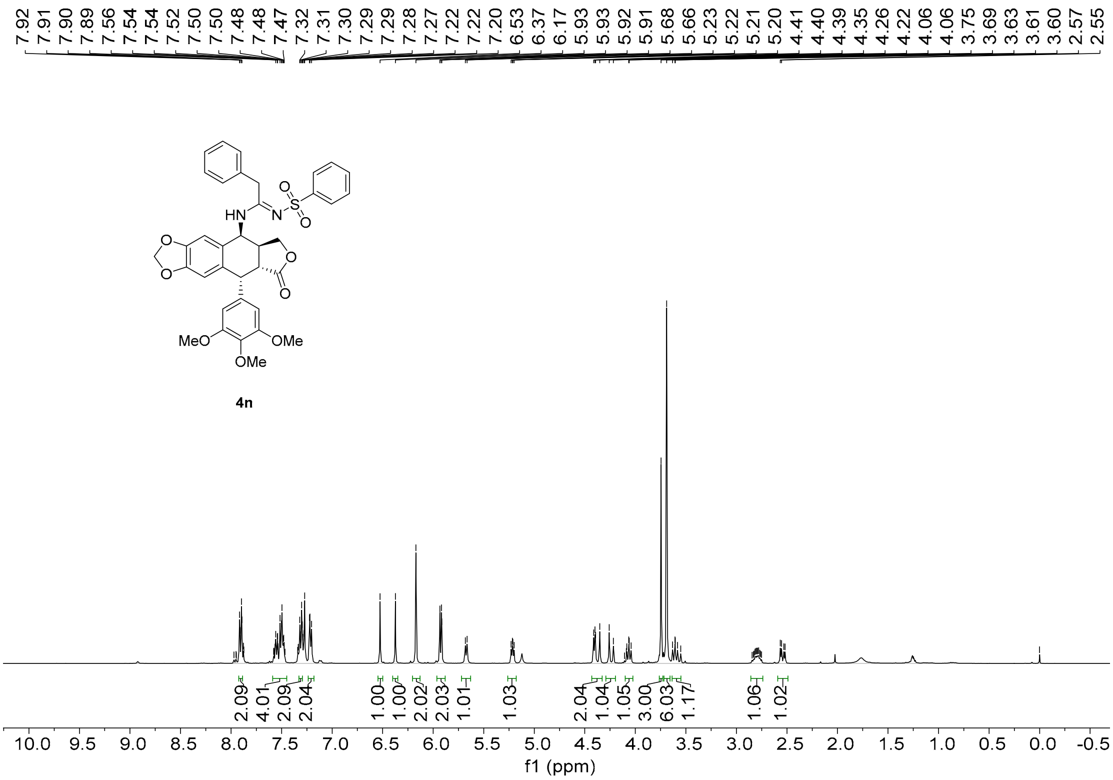

Figure S28 ^{13}C NMR spectrum of compound **4n** ($CDCl_3$)

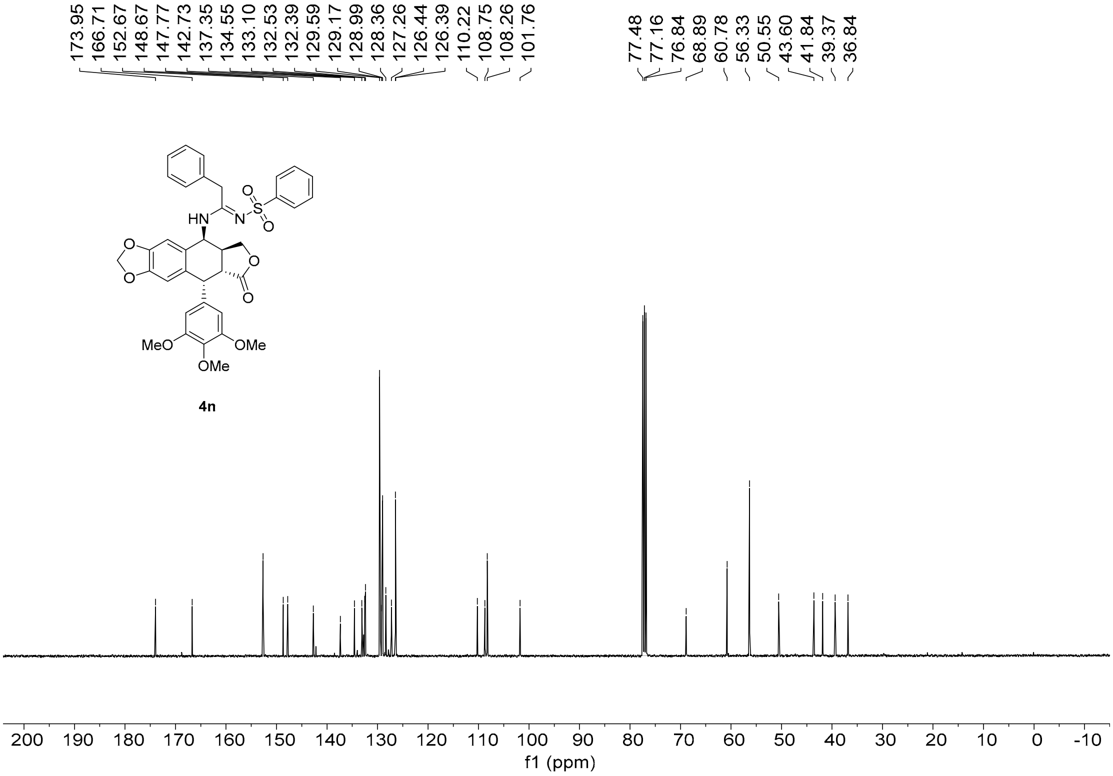

Figure S29 ^{1}H NMR spectrum of compound **4o** ($CDCl_3$)

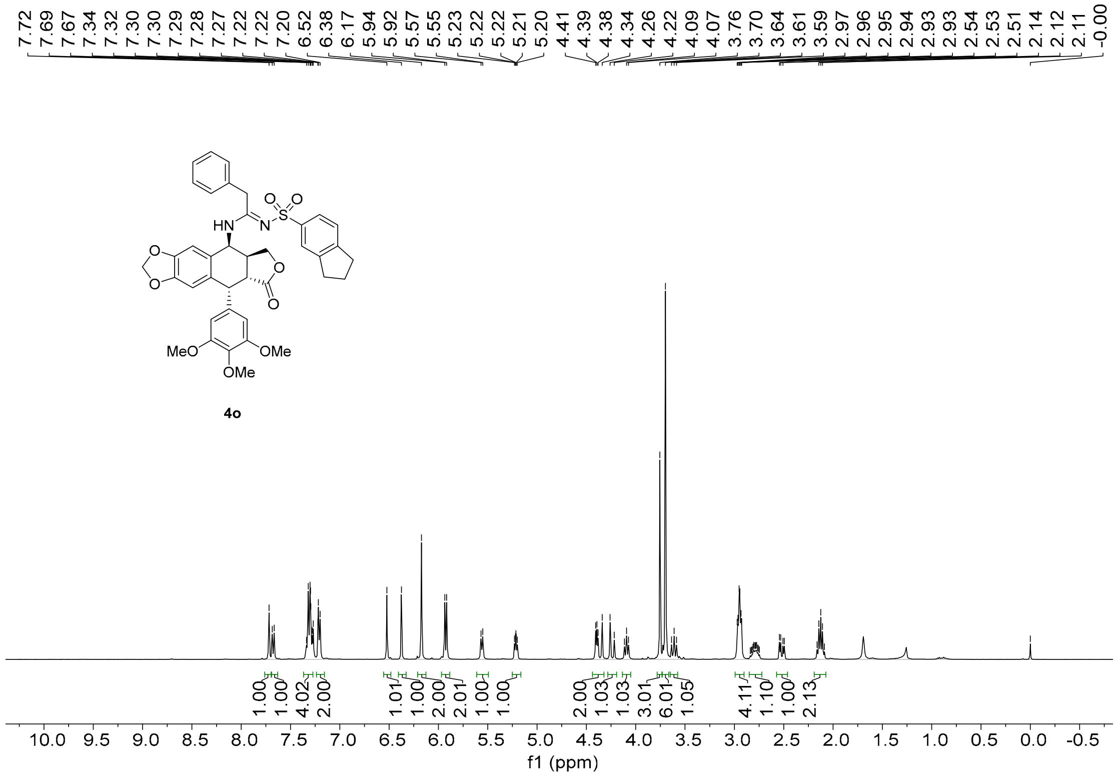

Figure S30 ^{13}C NMR spectrum of compound **4o** ($CDCl_3$)

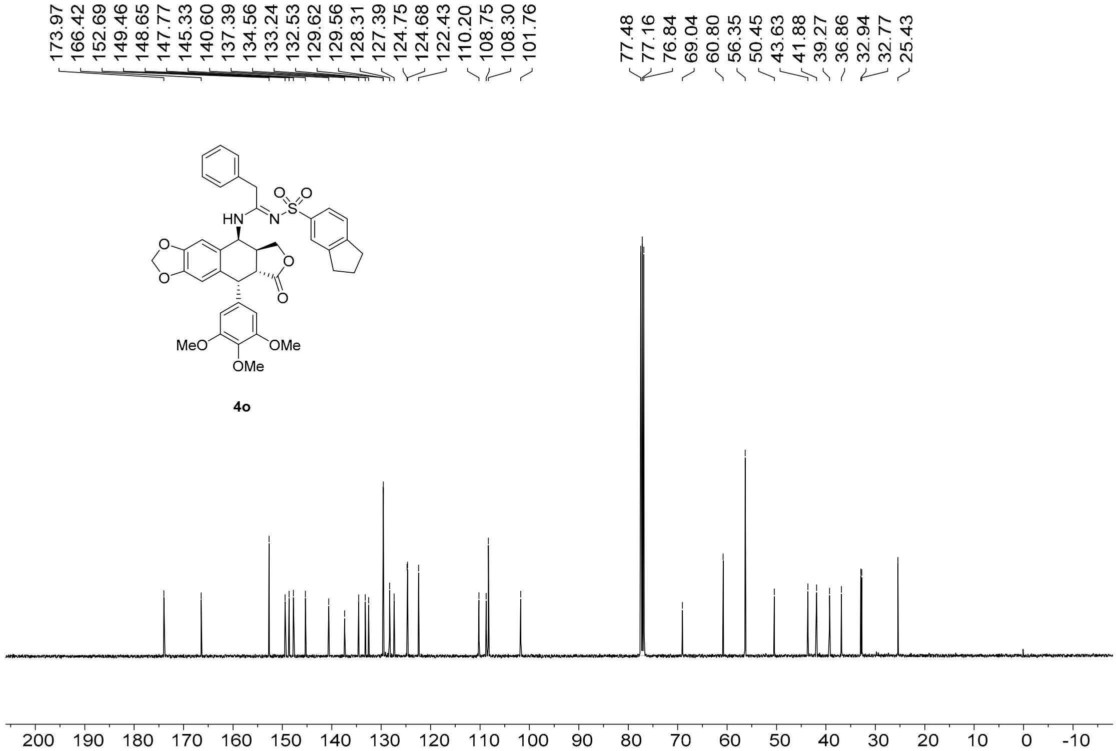

Figure S31 ^{1}H NMR spectrum of compound **4p** ($CDCl_3$)

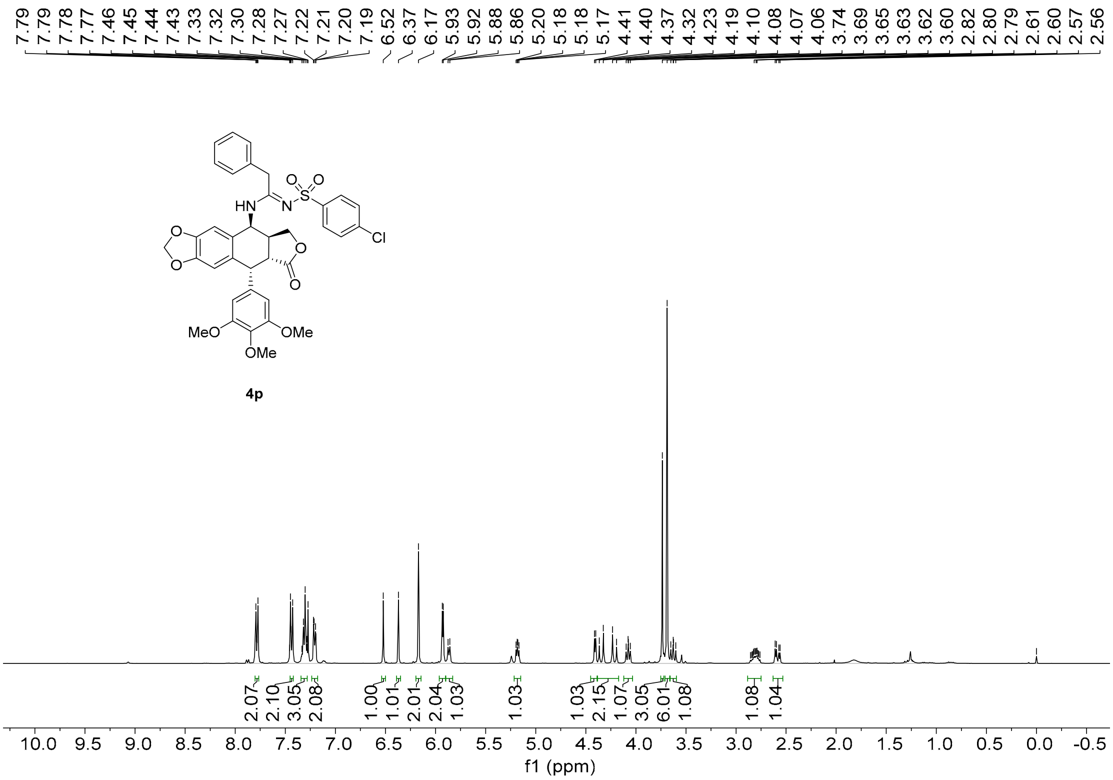

Figure S32 ^{13}C NMR spectrum of compound **4p** ($CDCl_3$)

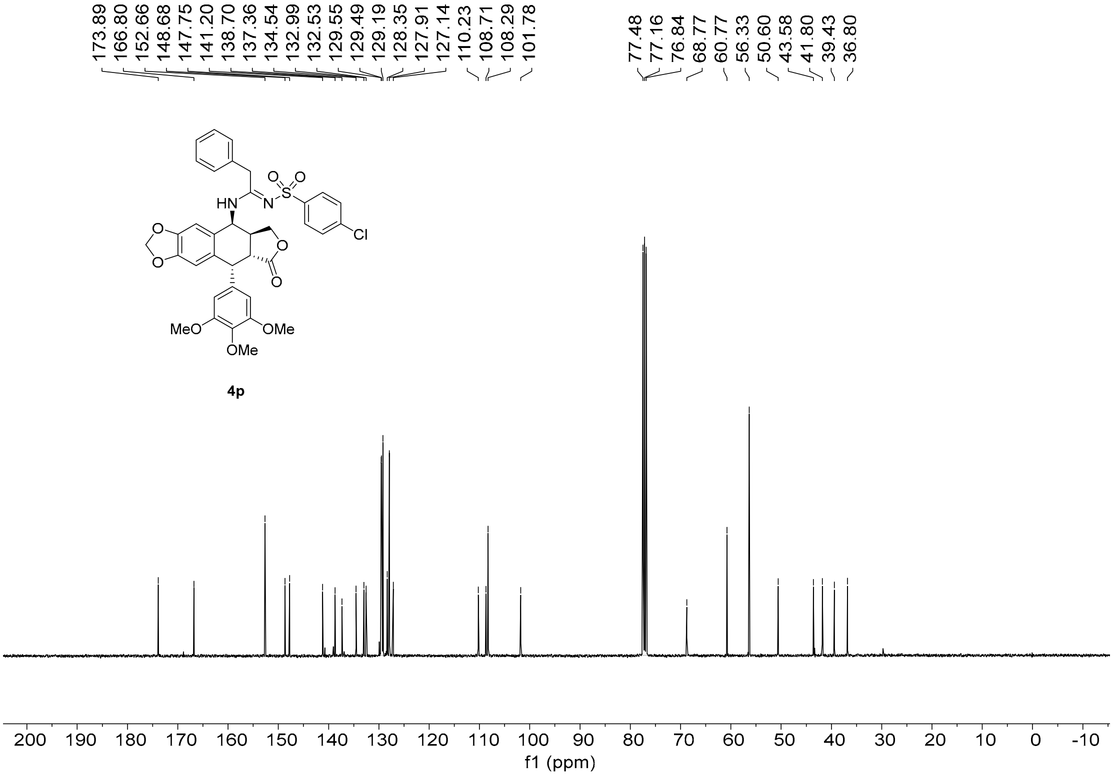

Figure S33 ^{1}H NMR spectrum of compound **4q** ($CDCl_3$)

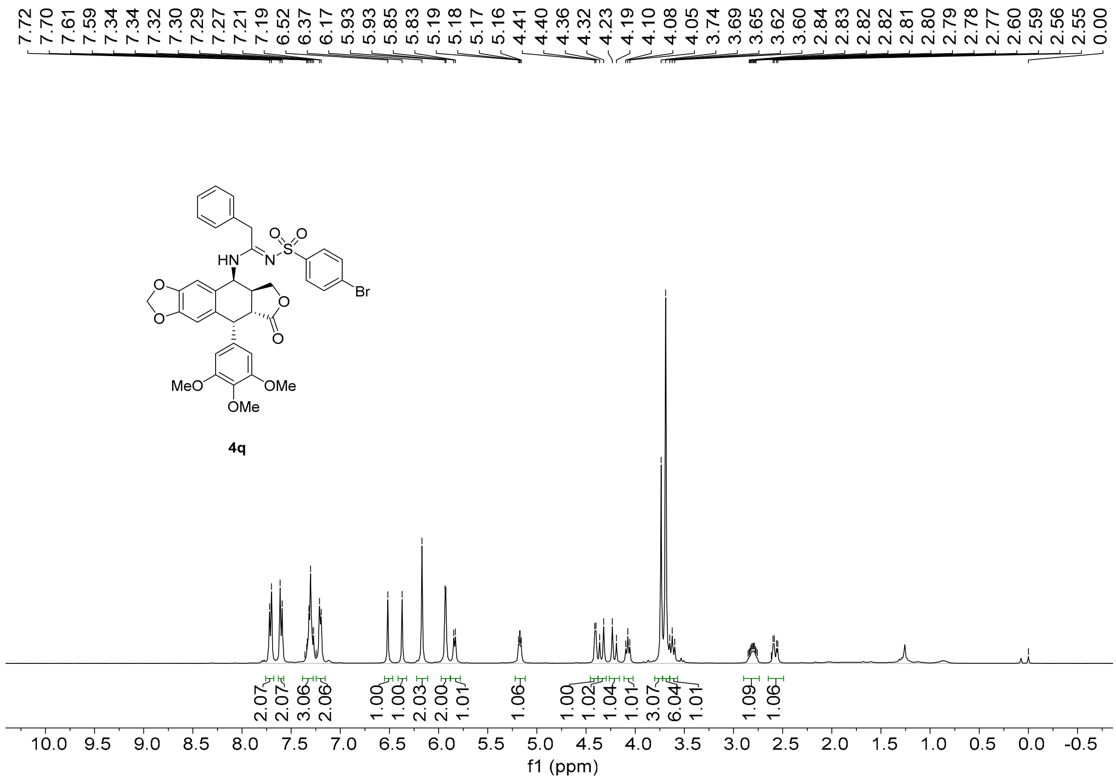

Figure S34 ^{13}C NMR spectrum of compound **4q** ($CDCl_3$)

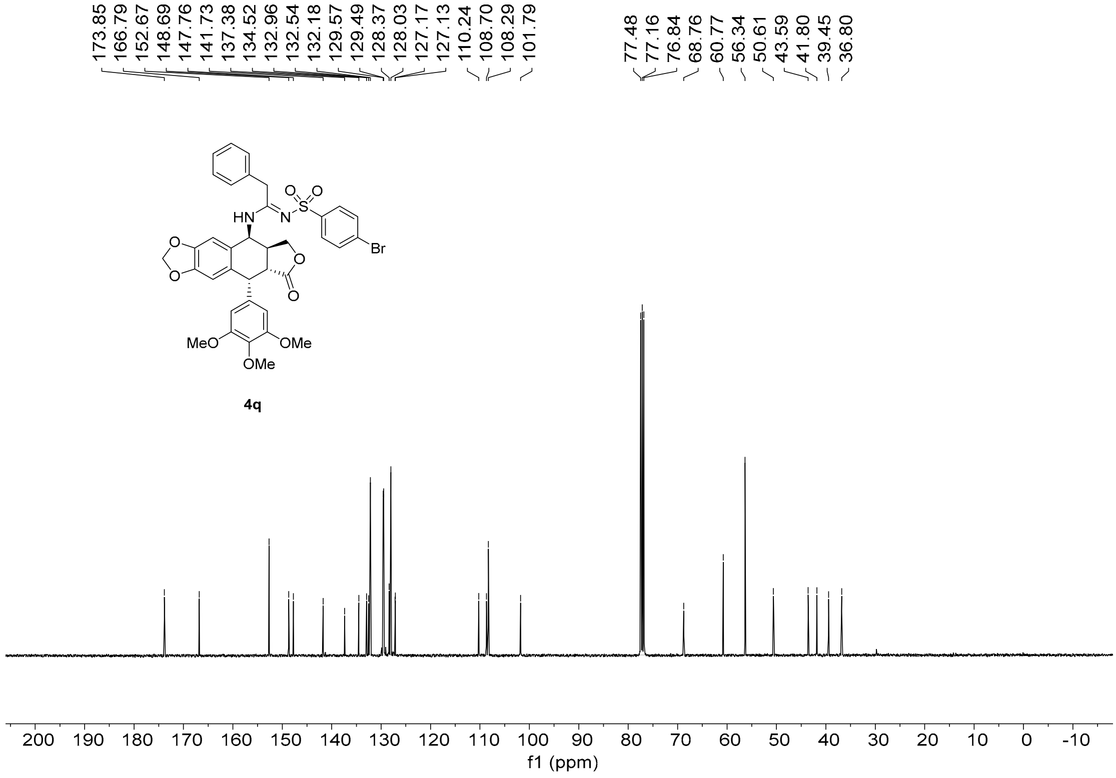

Figure S35 ^{1}H NMR spectrum of compound **4r** (($(CD_3)_2CO$))

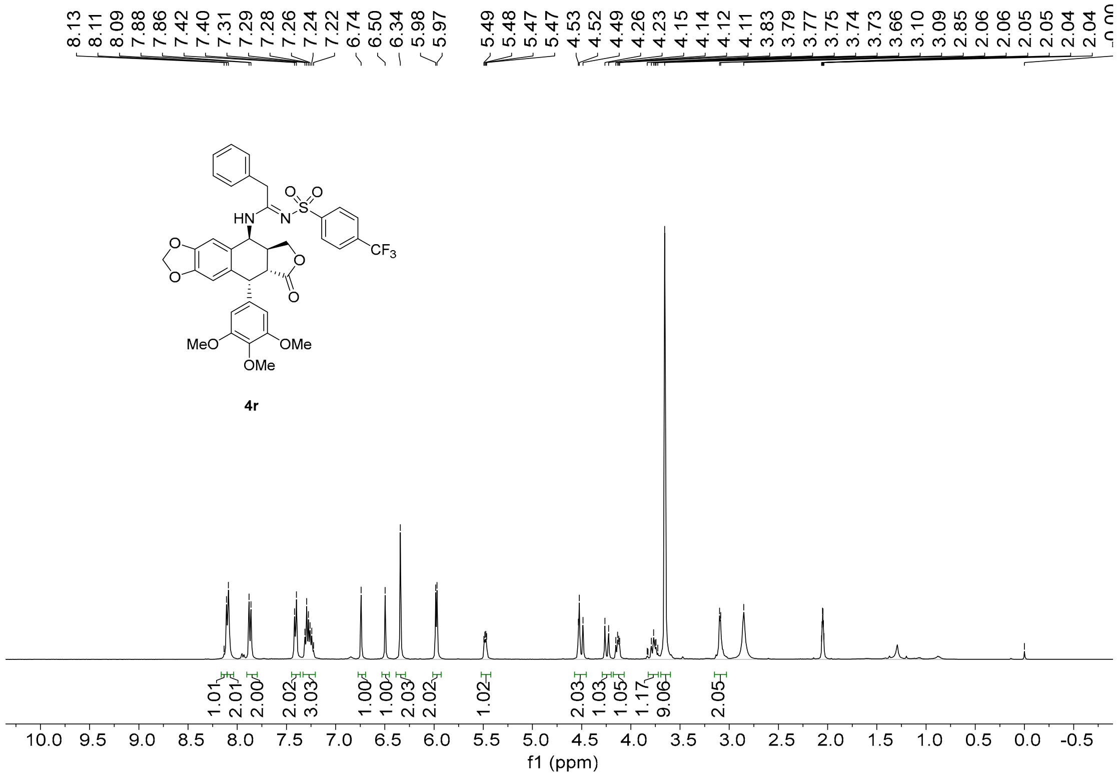

Figure S36 ^{13}C NMR spectrum of compound **4r** (($(CD_3)_2CO$))

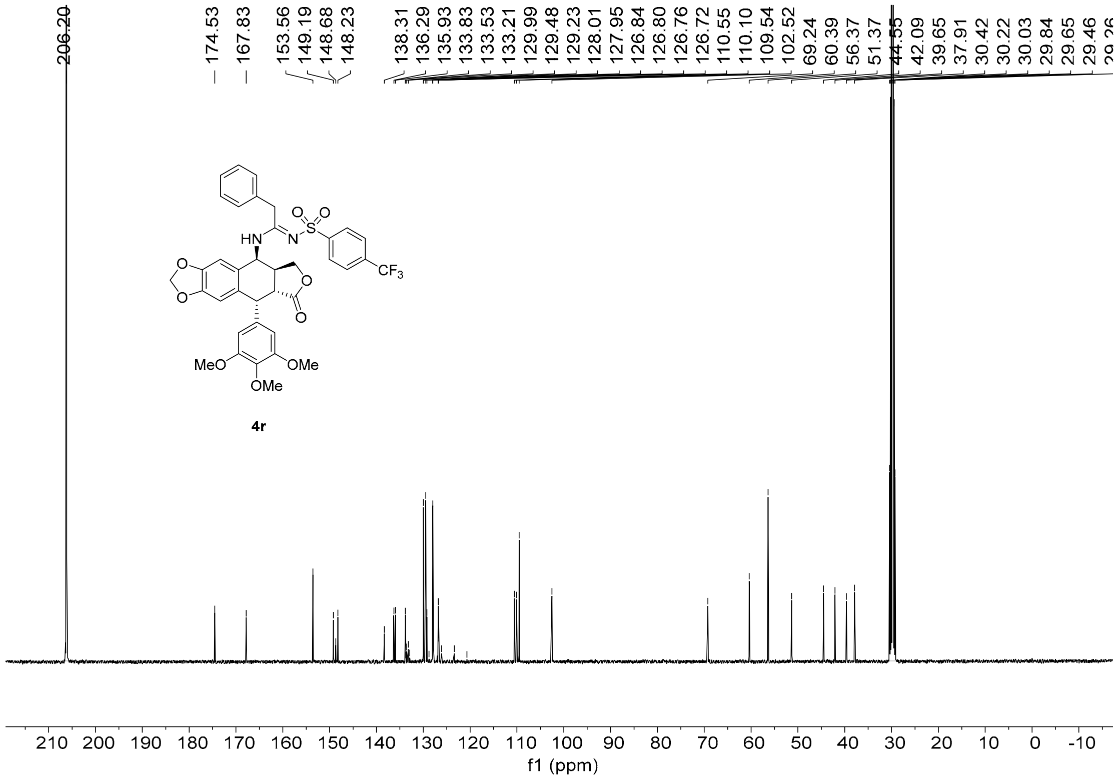

Figure S37 ^{1}H NMR spectrum of compound **4s** ($CDCl_3$)

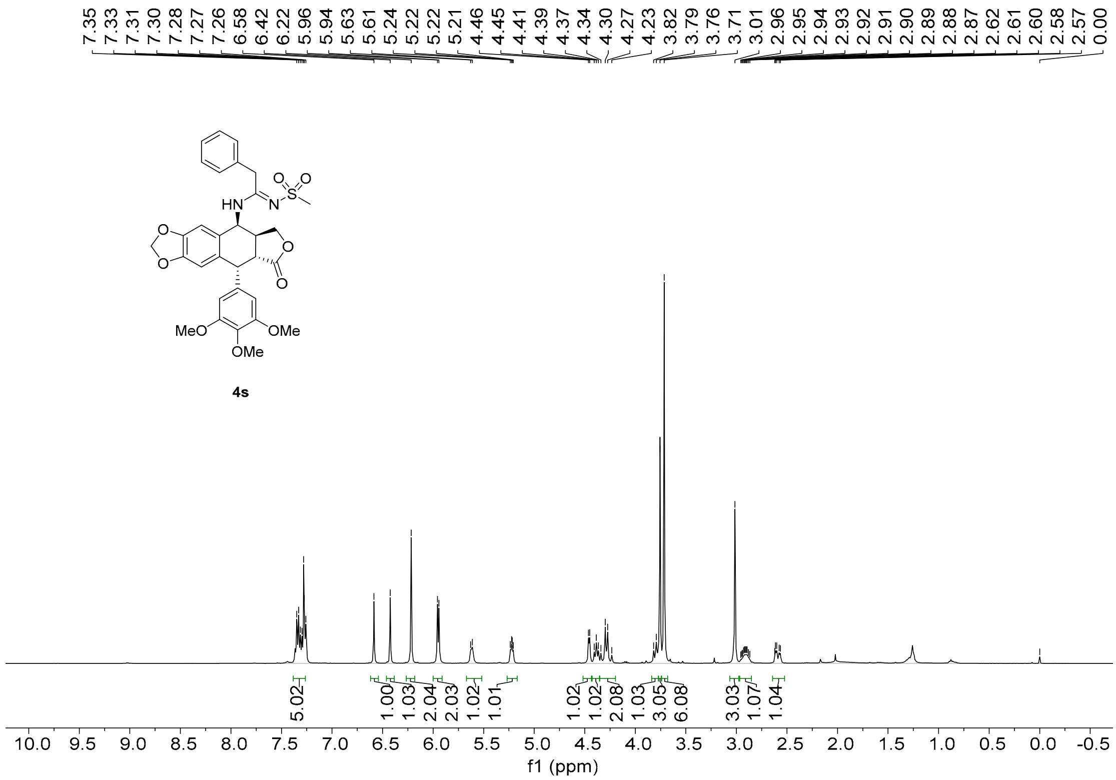

Figure S38 ^{13}C NMR spectrum of compound **4s** ($CDCl_3$)

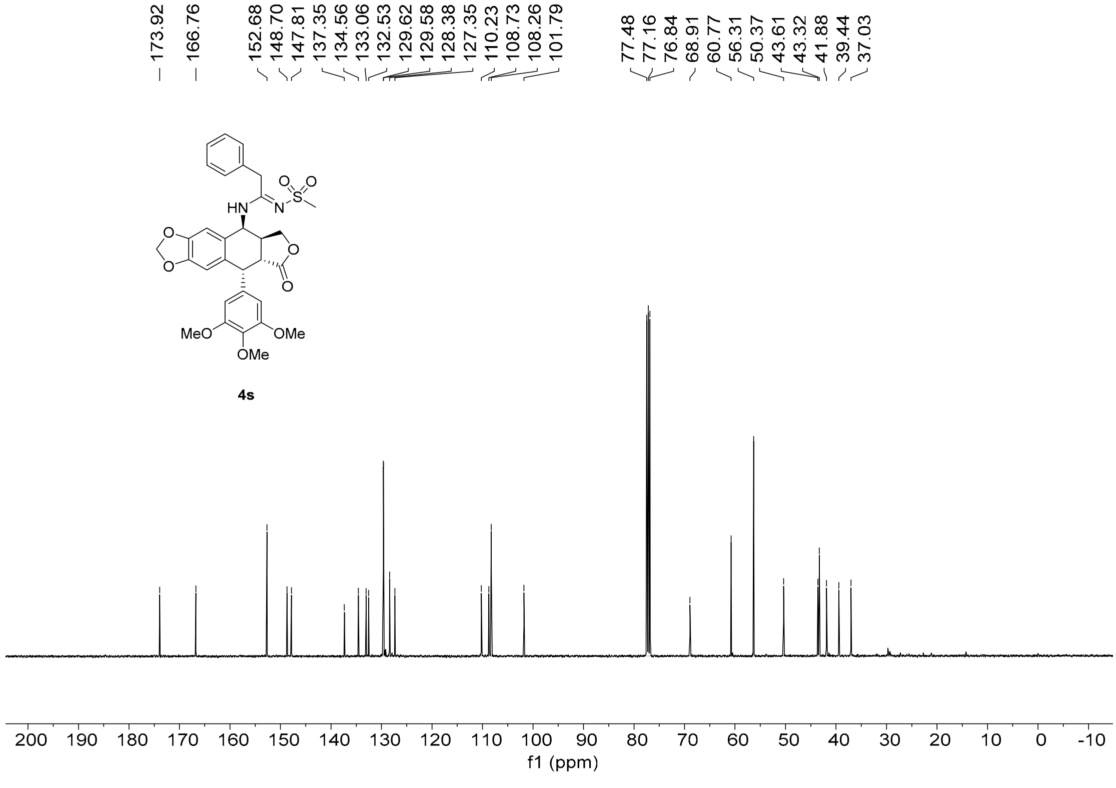

Figure S39 ^{1}H NMR spectrum of compound **4t** ($CDCl_3$)

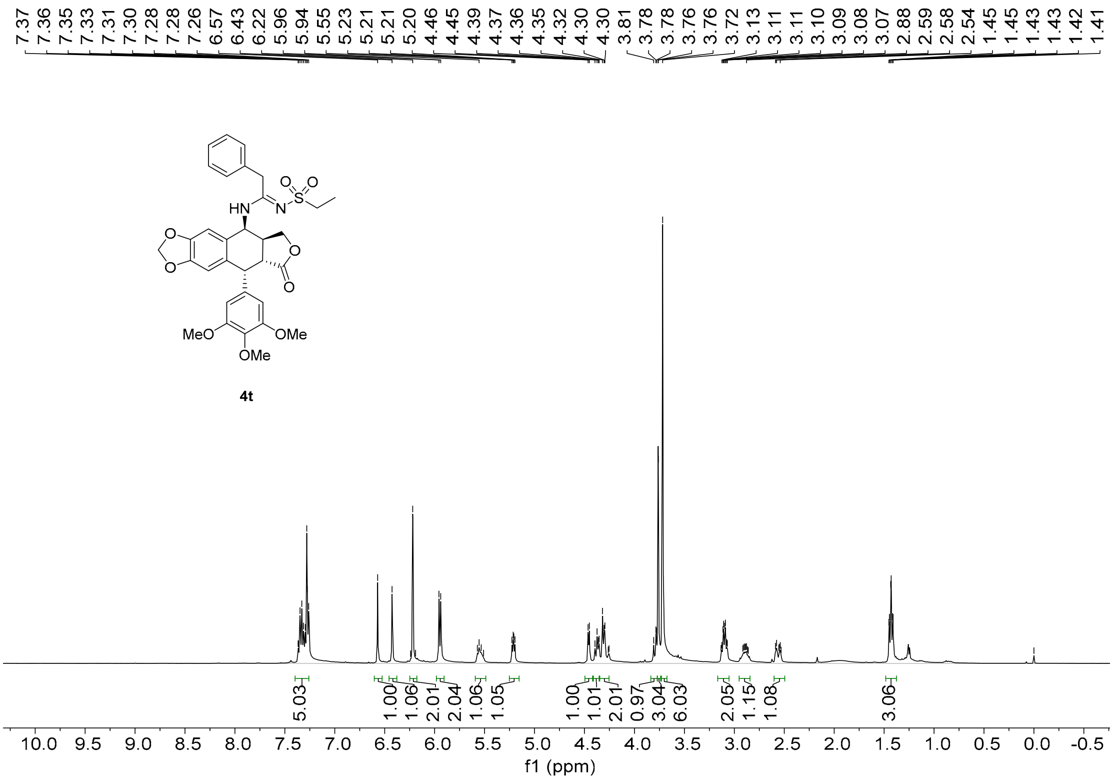

Figure S40 ^{13}C NMR spectrum of compound **4t** ($CDCl_3$)

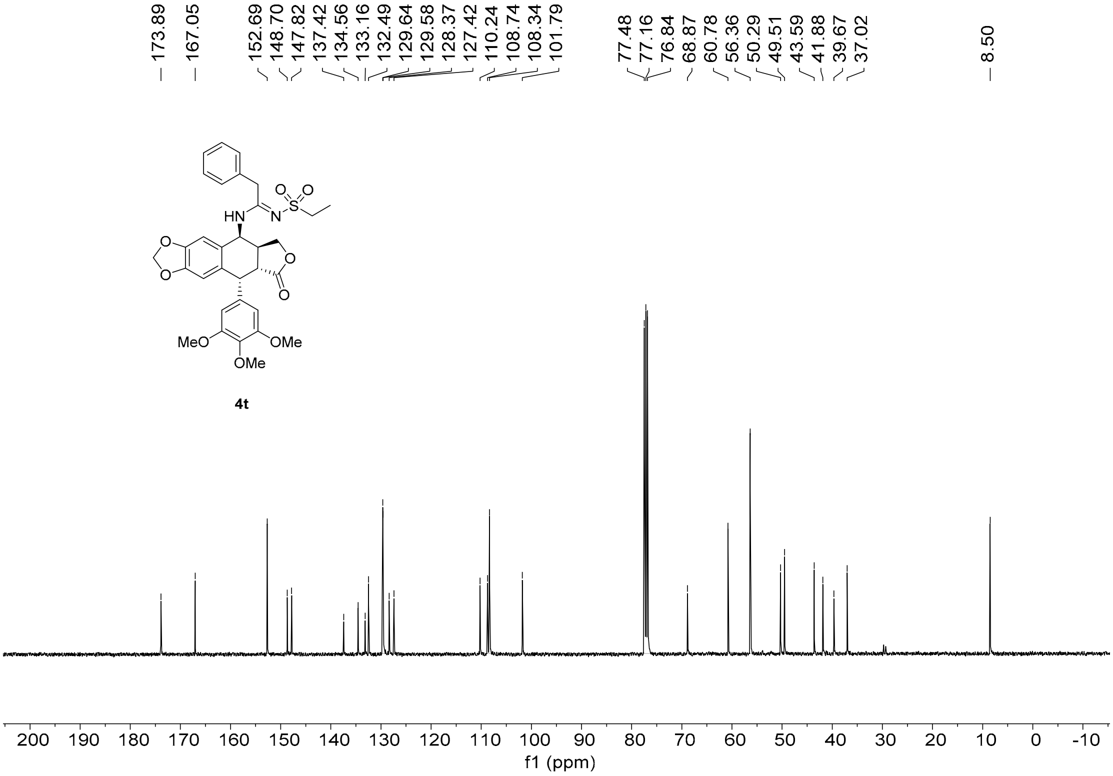

Figure S41 ^{1}H NMR spectrum of compound **4u** ($CDCl_3$)

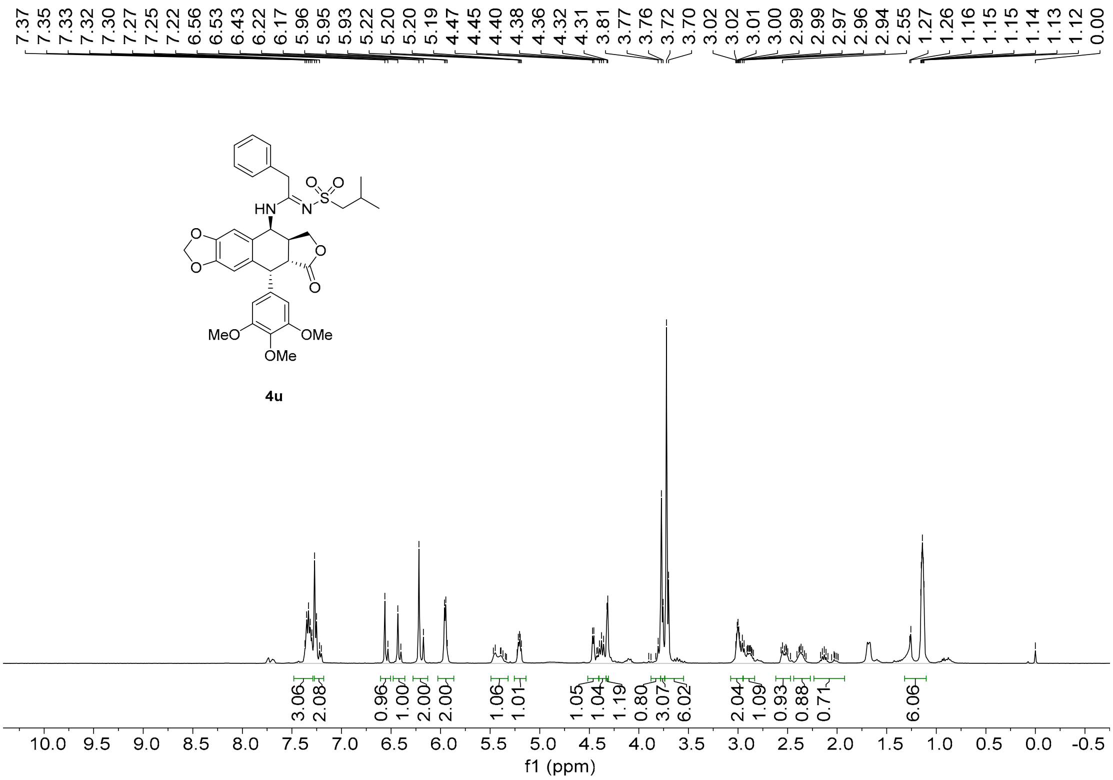

Figure S42 ^{13}C NMR spectrum of compound **4u** ($CDCl_3$)

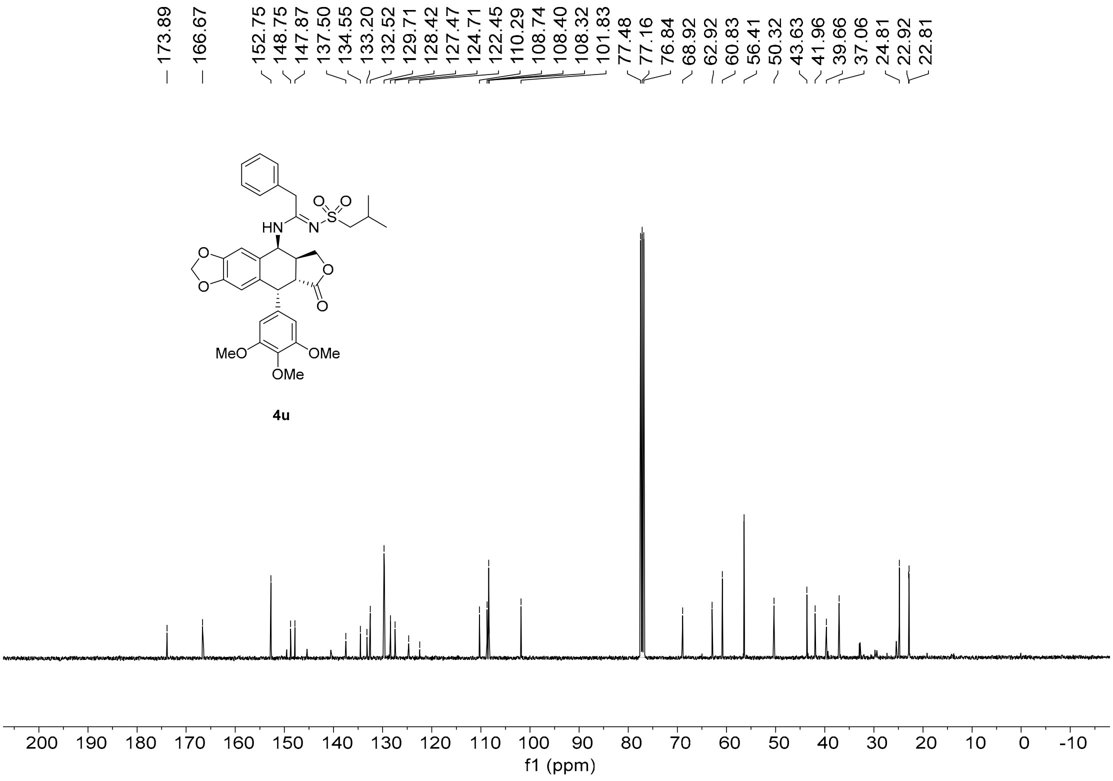

Figure S43 ^{1}H NMR spectrum of compound **4v** ($CDCl_3$)

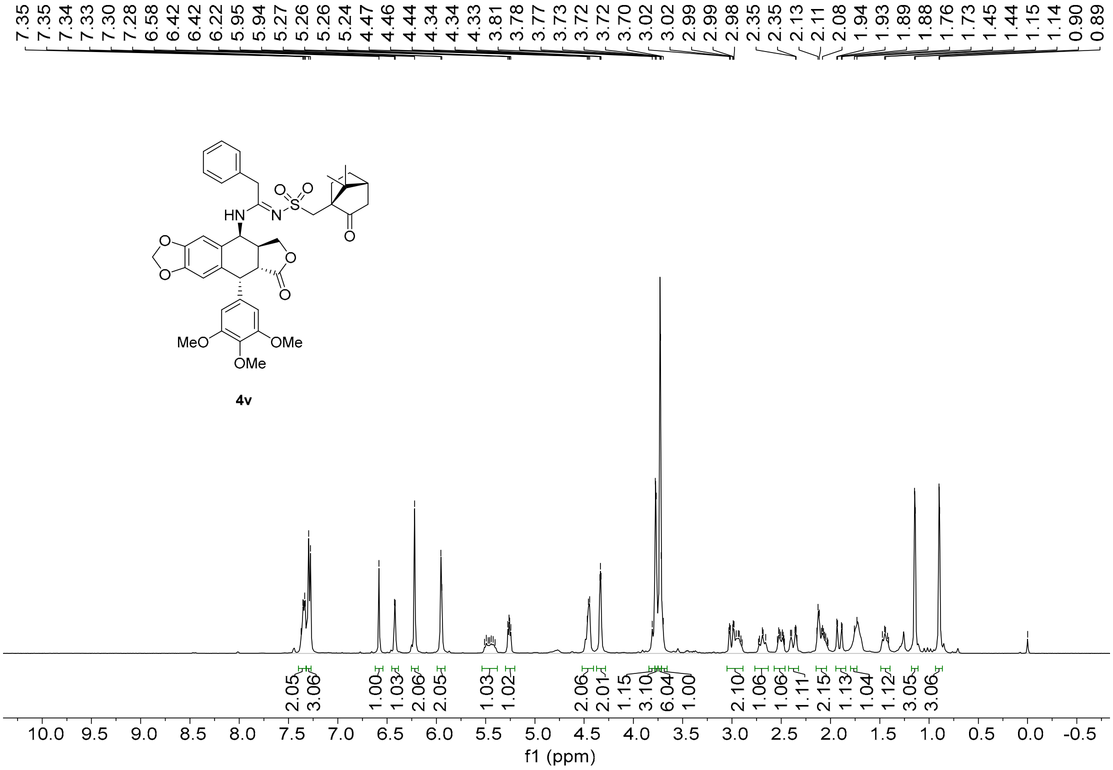

Figure S44 ^{13}C NMR spectrum of compound **4v** ($CDCl_3$)

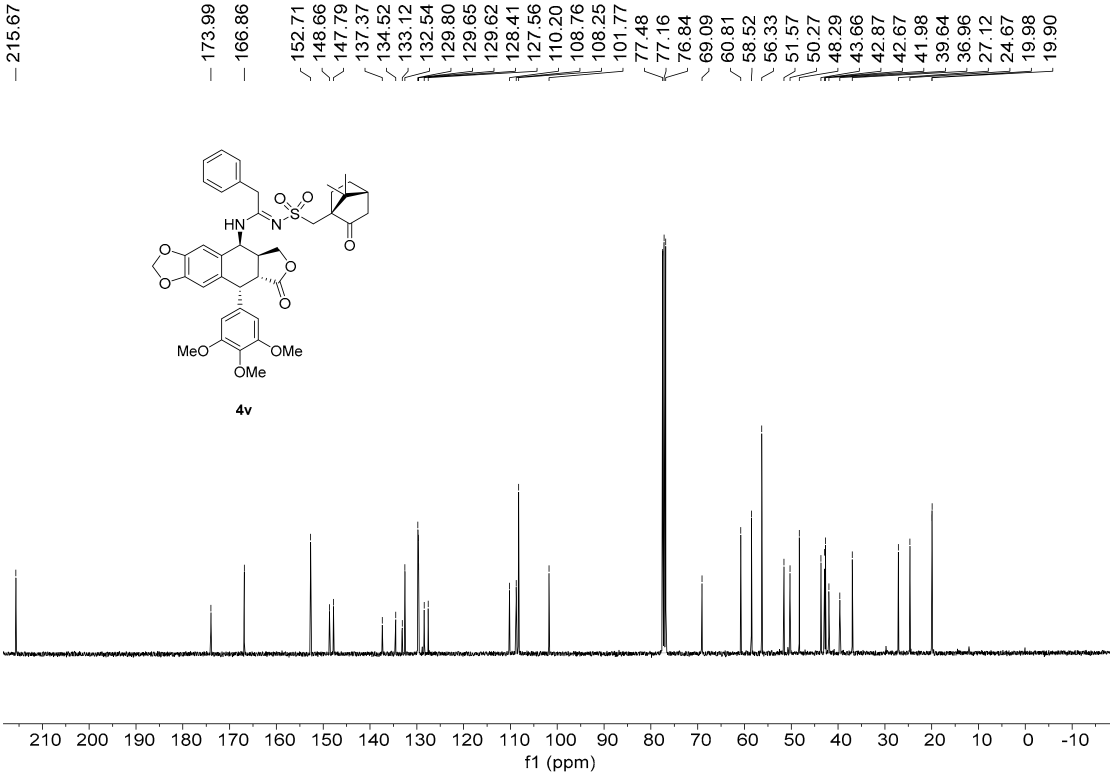

Figure S45 ^{1}H NMR spectrum of compound **5a** ($CDCl_3$)

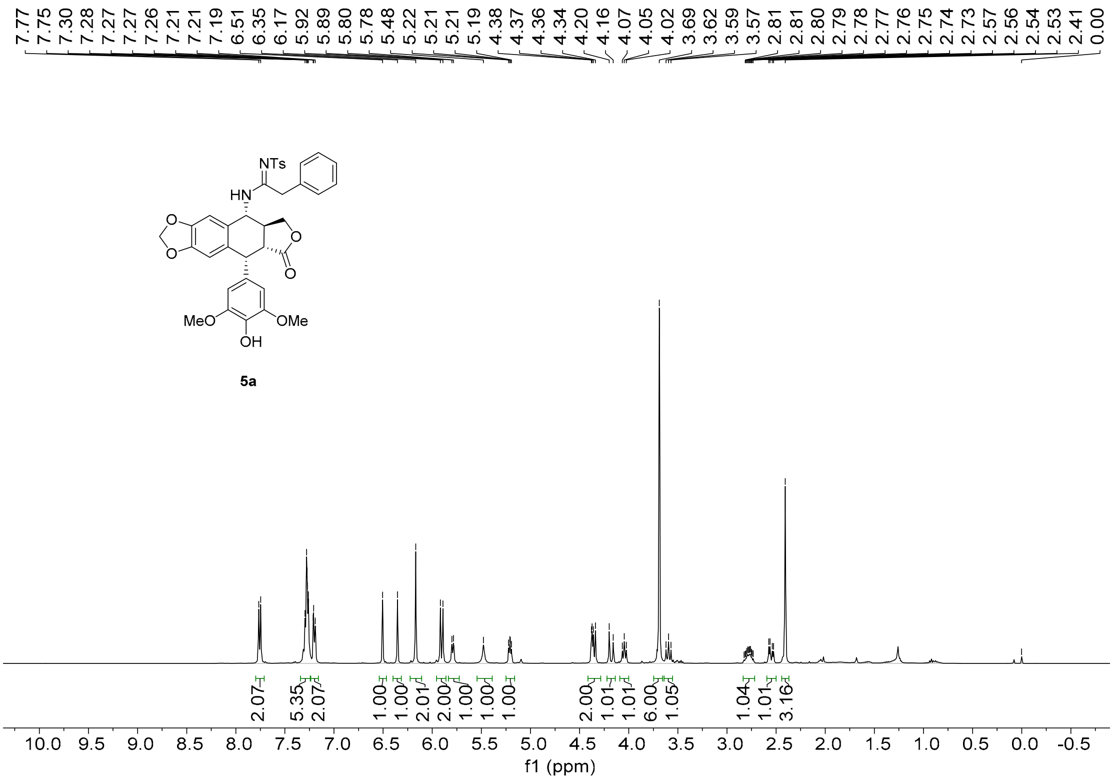

Figure S46 ^{13}C NMR spectrum of compound **5a** ($CDCl_3$)

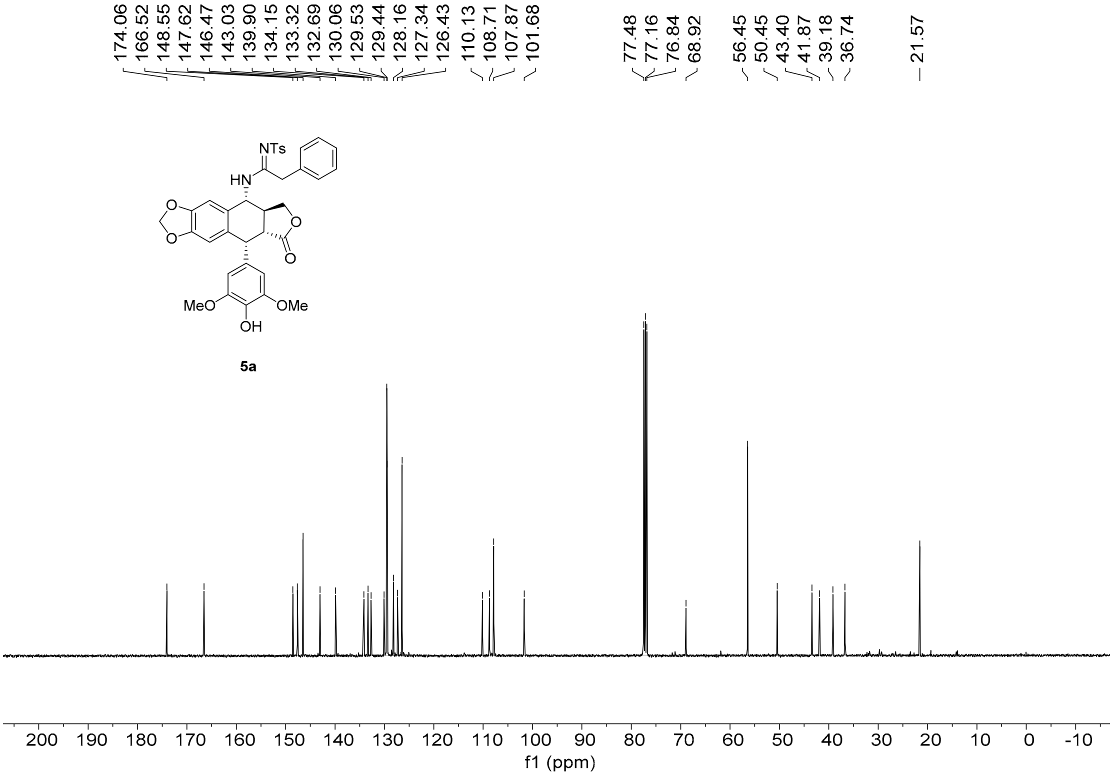

Figure S47 ^{1}H NMR spectrum of compound **5b** ($(CD_3)_2CO$)

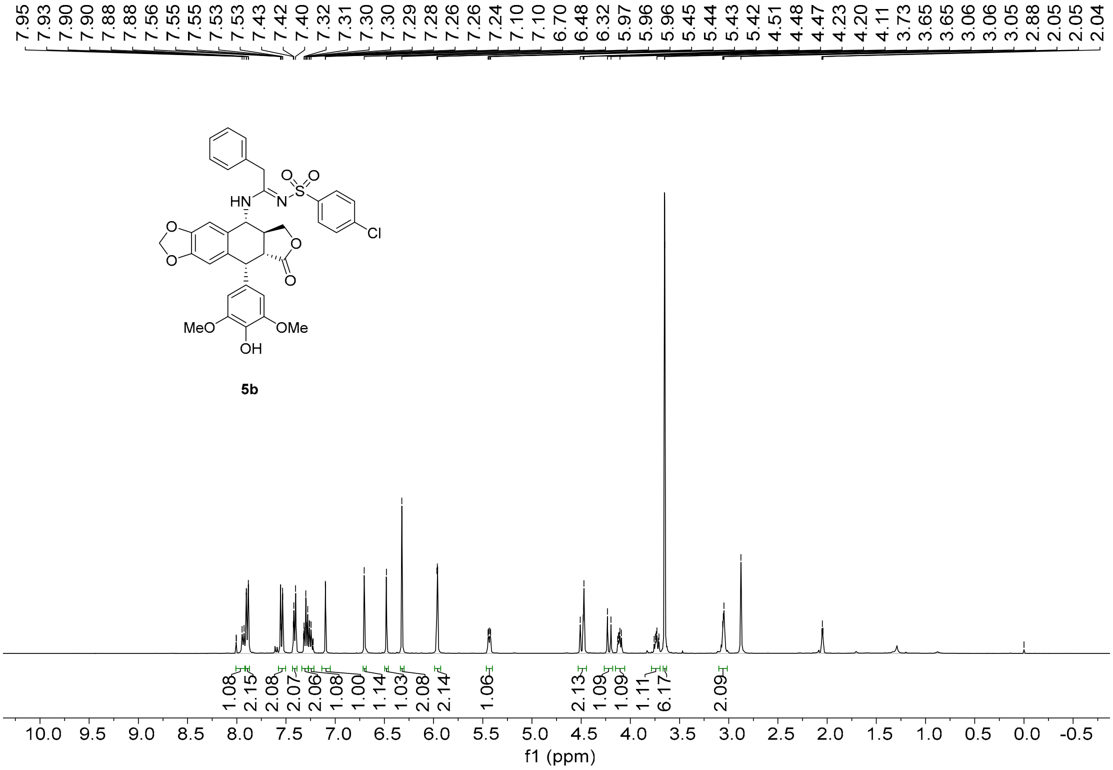

Figure S48 ^{13}C NMR spectrum of compound **5b** ($(CD_3)_2CO$)

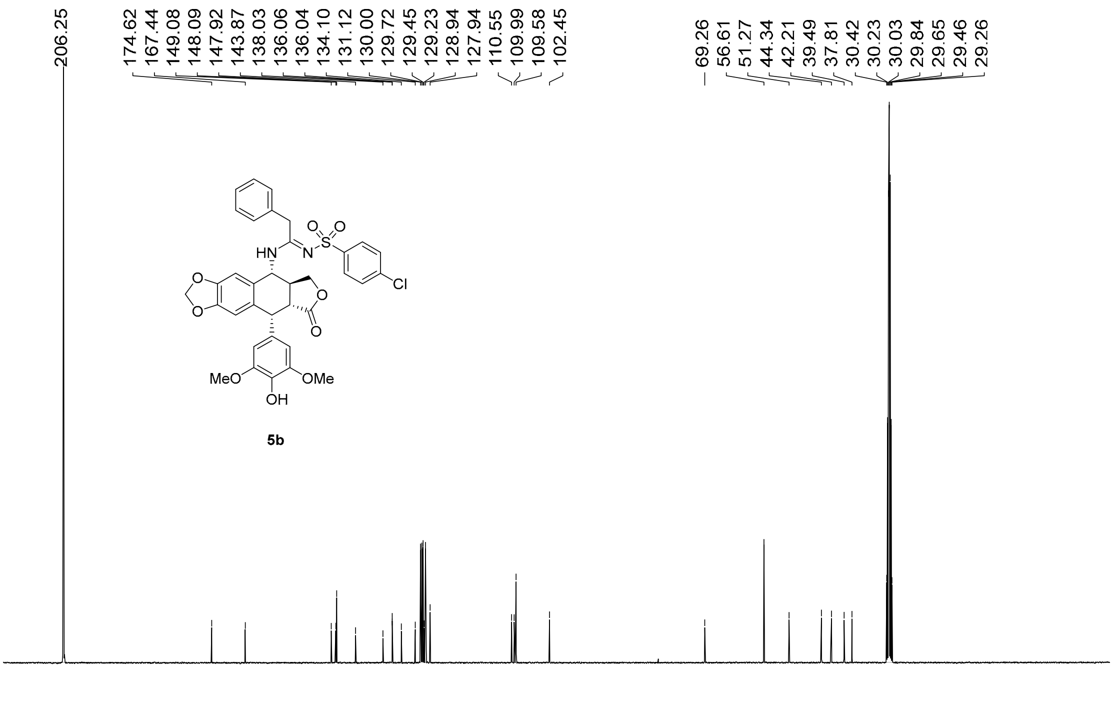

Figure S49 ^{1}H NMR spectrum of compound **5c** ($(CD_3)_2CO$)

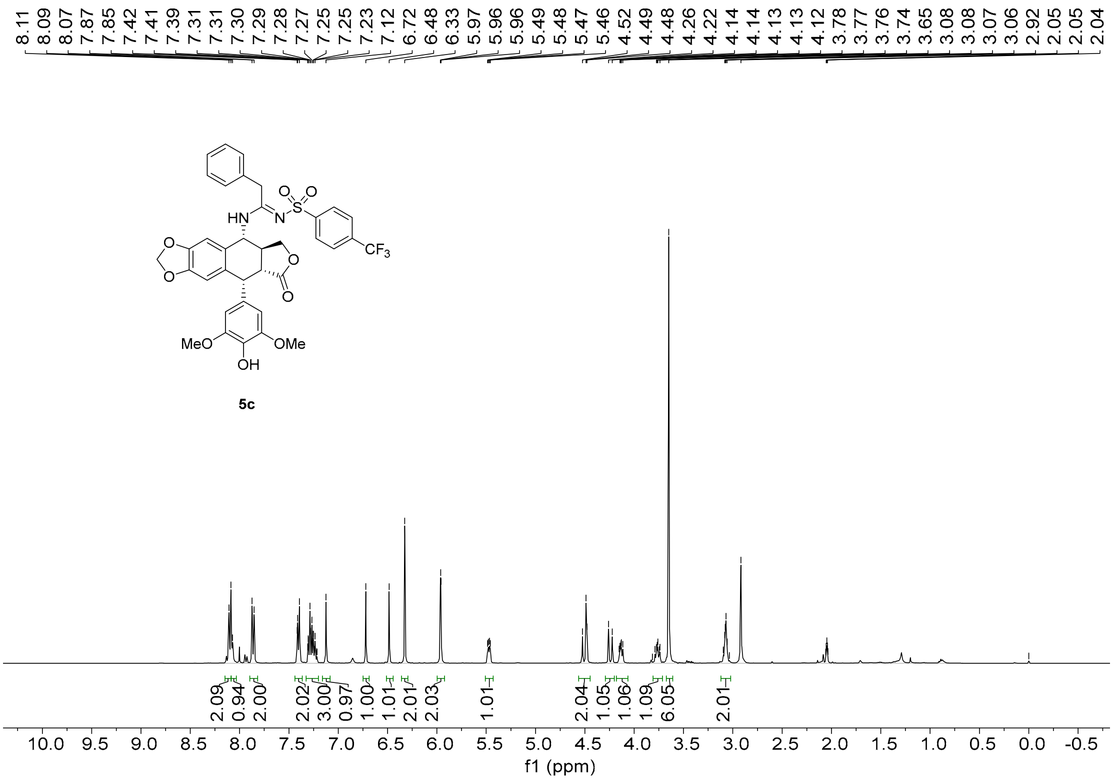

Figure S50 ^{13}C NMR spectrum of compound **5c** ($(CD_3)_2CO$)

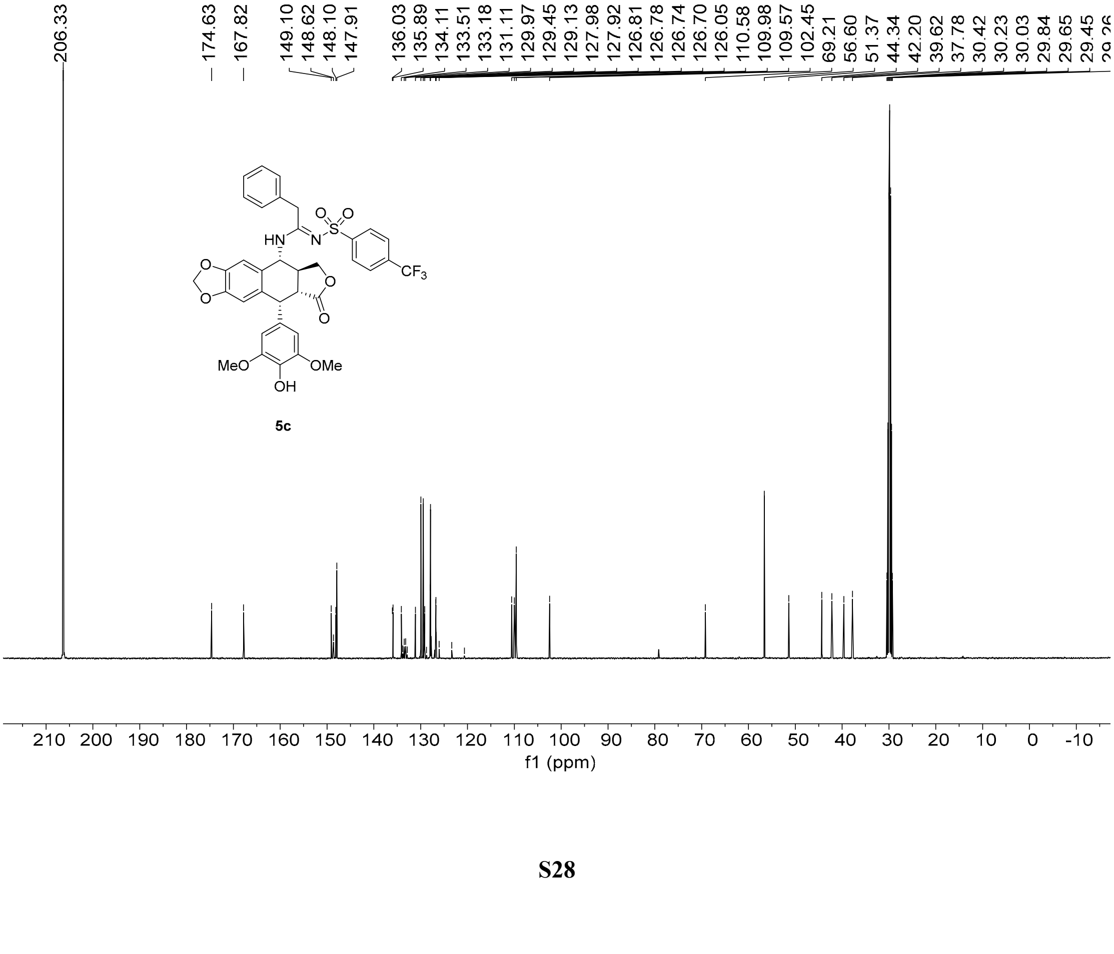

Figure S51 ^{1}H NMR spectrum of compound **5d** ($(CD_3)_2CO$)

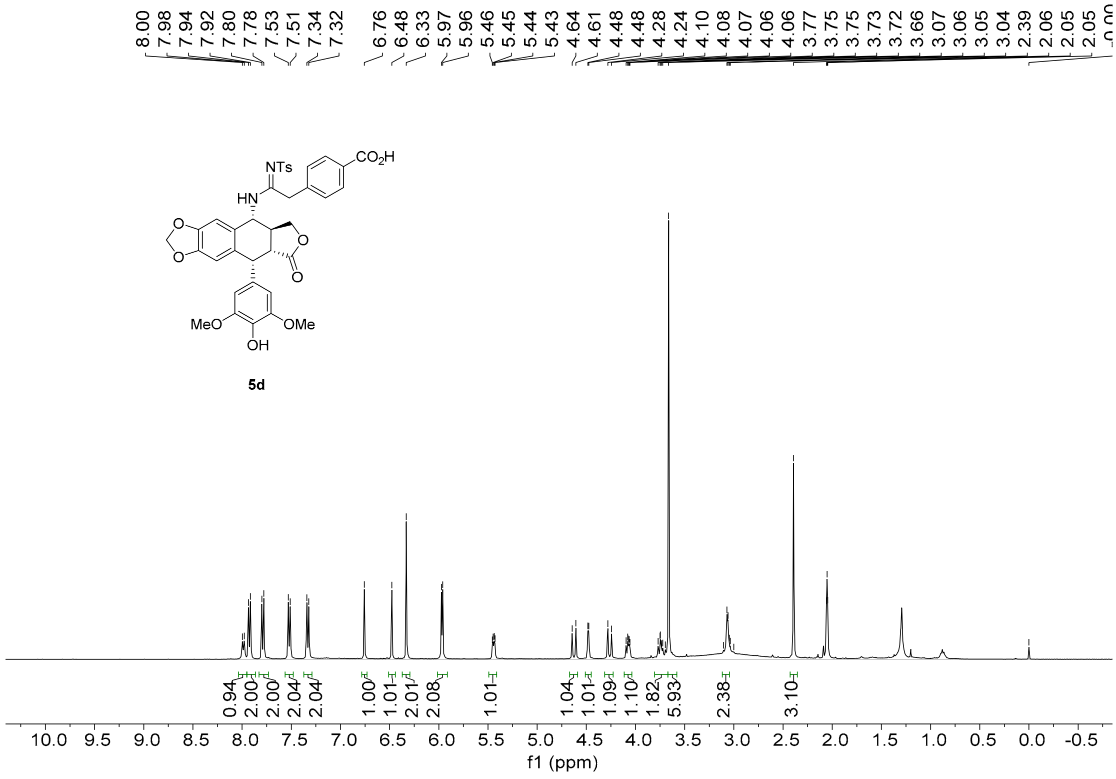

Figure S52 ^{13}C NMR spectrum of compound **5d** ($(CD_3)_2CO$)

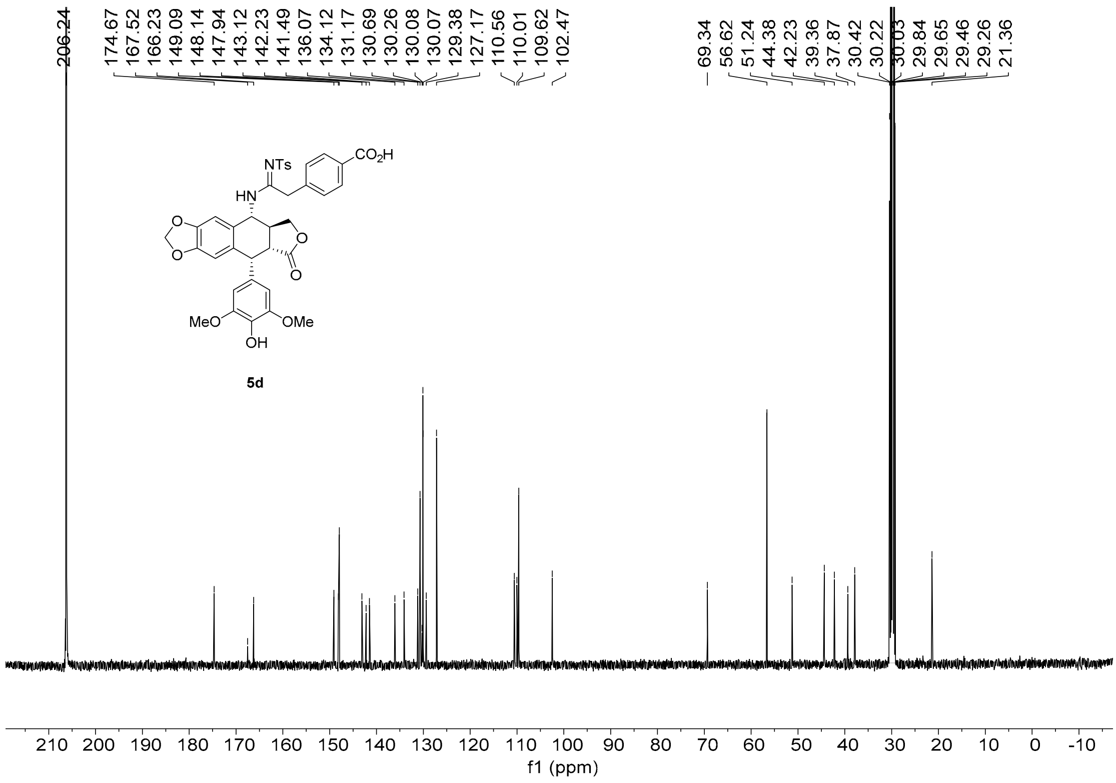

Figure S53 ^{1}H NMR spectrum of compound **5e** ($CDCl_3$)

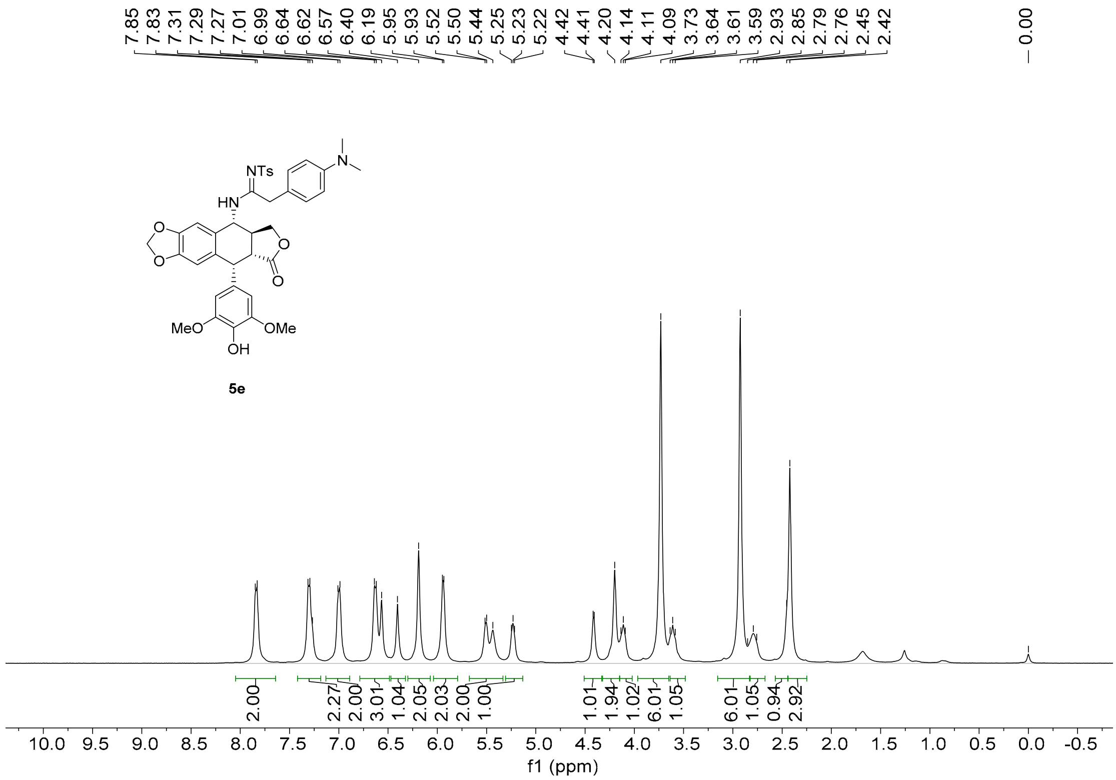

Figure S54 ^{13}C NMR spectrum of compound **5e** ($CDCl_3$)

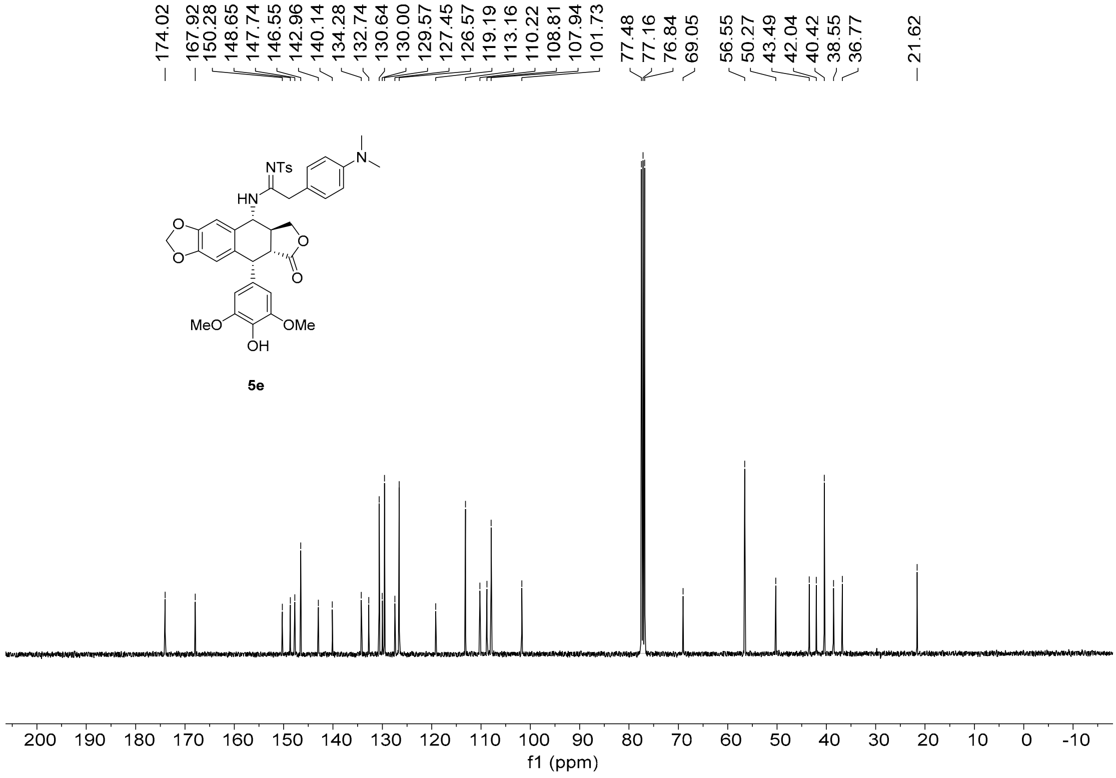

Figure S55 ^{1}H NMR spectrum of compound **5f** ($(CD_3)_2CO$)

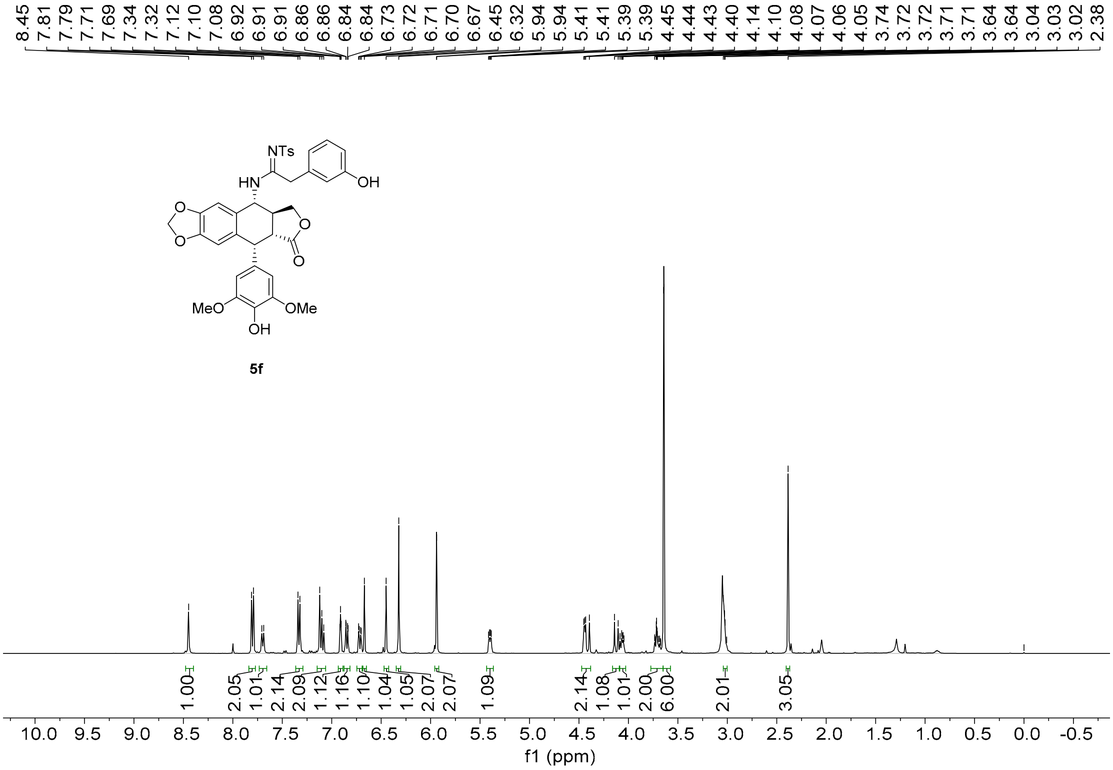

Figure S56 ^{13}C NMR spectrum of compound **5f** ($(CD_3)_2CO$)

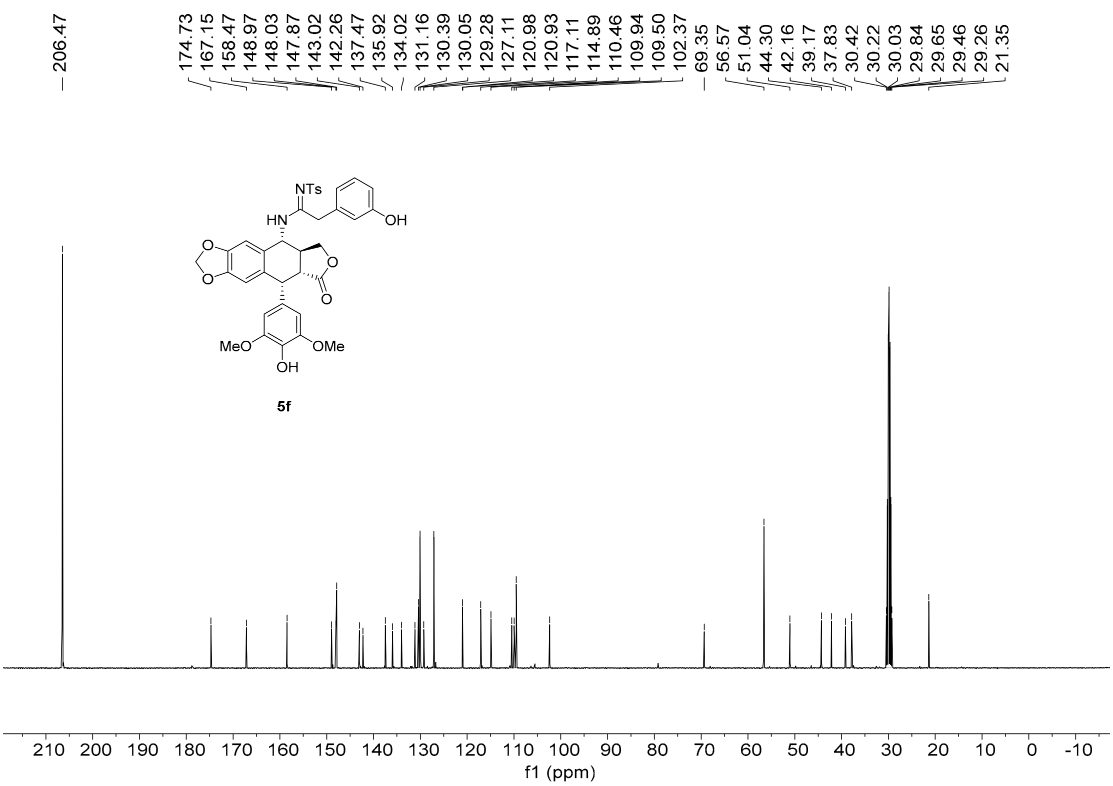

Supplement: Supplementary file 1 [file molecules-27-00220-s001.zip › molecules-1522867-supplementary.pdf]
